# Targeted deletion of CD244 on monocytes promotes differentiation into anti-tumorigenic macrophages and potentiates PD-L1 blockade in melanoma

**DOI:** 10.1186/s12943-024-01936-w

**Published:** 2024-02-29

**Authors:** Jeongsoo Kim, Tae-Jin Kim, Sehyun Chae, Hyojeong Ha, Yejin Park, Sunghee Park, Chul Joo Yoon, Seon Ah Lim, Hyemin Lee, Jiyoung Kim, Jungwon Kim, Kyungtaek Im, Kyunghye Lee, Jeongmin Kim, Daham Kim, Eunju Lee, Min Hwa Shin, Serk In Park, Inmoo Rhee, Keehoon Jung, Jeewon Lee, Keun Hwa Lee, Daehee Hwang, Kyung-Mi Lee

**Affiliations:** 1 Department of Biochemistry and Molecular biology, College of Medicine, Korea University, Seoul, 02841 South Korea; 2 Division of Radiation Biomedical Research, Korea Institute of Radiological and Medical Sciences, Seoul, 01812 South Korea; 3 Division of Chemical Engineering and Bioengineering, College of Art, Culture and Engineering, Kangwon National University, Chuncheon, 24341 South Korea; 4 Department of Chemical and Biological Engineering, College of Engineering, Korea University, Seoul, 02841 South Korea; 5 Department of Life Science, Ewha Womans University, Seoul, 03760 South Korea; 6 Department of Integrative Bioscience and Biotechnology, Sejong University, Seoul, 05006 South Korea; 7 Department of Biomedical Sciences, Department of Anatomy and Cell Biology, Seoul National University College of Medicine, Seoul, 03080 Republic of Korea; 8 Department of Microbiology, College of Medicine, Hanyang University, Seoul, 04763 South Korea; 9 School of Biological Sciences, Seoul National University, Seoul, 08826 South Korea; 10 Present Address: Immune Research Institute, Seegene Medical Foundation, Seoul, 04805 South Korea

**Keywords:** CD244, Macrophages, Differentiation, Macrophage-based cell therapy, Melanoma, Immune checkpoint blockade

## Abstract

**Background:**

In the myeloid compartment of the tumor microenvironment, CD244 signaling has been implicated in immunosuppressive phenotype of monocytes. However, the precise molecular mechanism and contribution of CD244 to tumor immunity in monocytes/macrophages remains elusive due to the co-existing lymphoid cells expressing CD244.

**Methods:**

To directly assess the role of CD244 in tumor-associated macrophages, monocyte-lineage-specific CD244-deficient mice were generated using cre-lox recombination and challenged with B16F10 melanoma. The phenotype and function of tumor-infiltrating macrophages along with antigen-specific CD8 T cells were analyzed by flow cytometry and single cell RNA sequencing data analysis, and the molecular mechanism underlying anti-tumorigenic macrophage differentiation, antigen presentation, phagocytosis was investigated ex vivo. Finally, the clinical feasibility of CD244-negative monocytes as a therapeutic modality in melanoma was confirmed by adoptive transfer experiments.

**Results:**

CD244^fl/fl^LysM^cre^ mice demonstrated a significant reduction in tumor volume (61% relative to that of the CD244^fl/fl^ control group) 14 days after tumor implantation. Within tumor mass, CD244^fl/fl^LysM^cre^ mice also showed higher percentages of Ly6C^low^ macrophages, along with elevated gp100^+^IFN-γ^+^ CD8 T cells. Flow cytometry and RNA sequencing data demonstrated that ER stress resulted in increased CD244 expression on monocytes. This, in turn, impeded the generation of anti-tumorigenic Ly6C^low^ macrophages, phagocytosis and MHC-I antigen presentation by suppressing autophagy pathways. Combining anti-PD-L1 antibody with CD244^−/−^ bone marrow-derived macrophages markedly improved tumor rejection compared to the anti-PD-L1 antibody alone or in combination with wild-type macrophages. Consistent with the murine data, transcriptome analysis of human melanoma tissue single-cell RNA-sequencing dataset revealed close association between CD244 and the inhibition of macrophage maturation and function. Furthermore, the presence of CD244-negative monocytes/macrophages significantly increased patient survival in primary and metastatic tumors.

**Conclusion:**

Our study highlights the novel role of CD244 on monocytes/macrophages in restraining anti-tumorigenic macrophage generation and tumor antigen-specific T cell response in melanoma. Importantly, our findings suggest that CD244-deficient macrophages could potentially be used as a therapeutic agent in combination with immune checkpoint inhibitors. Furthermore, CD244 expression in monocyte-lineage cells serve as a prognostic marker in cancer patients.

**Supplementary Information:**

The online version contains supplementary material available at 10.1186/s12943-024-01936-w.

## Background

Immune exhaustion has been primarily centered on T cells, upregulating multiple checkpoint receptors including programmed cell death-1 (PD-1), cytotoxic T-lymphocyte-associated antigen-4 (CTLA-4), lymphocyte-activation gene-3 (LAG-3), T-cell immunoglobulin and mucin-domain containing-3 (TIM-3), T cell immunoreceptor with Ig and ITIM domains (TIGIT), CD160, and CD244 (SLAMF4, 2B4) within the immunosuppressive tumor microenvironment (TME) [1–4]. Accordingly, therapeutic antibodies targeting CTLA-4, PD-1, programmed cell death ligand-1(PD-L1), and LAG-3 have been developed to release the brakes imposed by checkpoint receptors in advanced cancers [5–8]. However, treatment responses remain suboptimal for many patients due to tumor heterogeneity, lack of immune cell infiltration, and impaired antigen presentation [9–13]. Accumulating data suggest that immunosuppressive phenotypes of monocytes and macrophages contribute to the immune exhaustion of T cells and NK cells in TME [14]. The immunosuppressive activities of monocyte-lineage cells are mediated by various mechanisms, including the production of reactive oxygen/nitrogen species, nutrients depletion, secretion of inhibitory cytokines, and expression of immune checkpoint ligands, contributing to the exhausted tumor-immune microenvironment that does not respond to immunotherapy [15].

CD244 belongs to the signaling lymphocyte activation molecule family (SLAMF) and has been shown to maintain the exhausted phenotype of T cells and NK cells within TME [9, 16–20]. Recent studies have revealed the expression of CD244 on monocyte-lineage cells, suggesting a positive correlation between the expression level of CD244 on tumor-infiltrating monocytes and their immunosuppressive phenotype, such as the expression of arginase-1 (ARG-1), interleukin-10 (IL-10), and transforming growth factor-β (TGF-β) [21]. However, the direct impact and mechanism of CD244 on monocytes and macrophages within TME have not been fully elucidated due to its broad expression on various immune cells, including CD8 T cells, NK cells, γδ T cells, dendritic cells (DCs), and neutrophils.

To directly assess the role of CD244 in immune dysfunction of monocyte-lineage cells, we generated mice with monocyte lineage-specific CD244 deficiency using cre-lox recombination (CD244^fl/fl^LysM^cre^). In vivo and ex vivo analysis of bone marrow-derived macrophages harvested from CD244^fl/fl^LysM^cre^ mice challenged with B16F10 melanoma demonstrated that the absence of CD244 not only enhanced Ly6C^low^ macrophage differentiation but also their anti-tumorigenic functions such as antigen presentation and phagocytosis. Consistent with the murine data, transcriptome analysis of human melanoma tissue single-cell RNA-sequencing dataset (SCP398 from single-cell portal) revealed close association between CD244 and the suppression of phagocytosis, antigen presentation, and autophagy. Furthermore, conducting cell type deconvolution analysis on bulk RNA-seq datasets from melanoma patients in the TCGA database unveiled that the presence of CD244-negative monocytes/macrophages was associated with a significant increase in patient survival, both in primary and metastatic tumors. Taken together, our findings highlight the role of CD244 as an immune checkpoint receptor that restrains adaptive immunity mediated by monocyte-lineage cells. Furthermore, our study provides practical evidence supporting the use of CD244-deficient macrophages as a therapeutic modality to enhance the efficacy of checkpoint blockade therapies.

## Methods

### Study design

This study was a controlled laboratory experiment using a mouse model and primarily aimed to identify the role of CD244 expressed on monocytes/macrophages in the TME. The sample size of the mouse tumor implantation experiments was determined to achieve a significance level greater than 95% using G-power software. No outliers were excluded from the experiments or analyses reported in this study. The endpoint of the mouse tumor growth experiment was determined according to the guidelines of the Institutional Animal Care and Use Committee of Korea University. For all studies, the number of samples and independently performed experiments are indicated in the figure legends. The sum of the data units represents the number of samples, which are depicted as individual values in the bar graphs. The experiments were not randomized. The investigators were not blinded to allocation during the experiments or outcome assessment.

### Mice

All animal experiments were approved by the Institutional Animal Care and Use Committee of Korea University (approval number: KUIACUC-2019-0101, KUIACUC-2022-0049) and followed their guidelines and regulations. Wild-type (WT) C57BL/6 mice were purchased from Orient Bio. Inc. (Seongnam-si, Korea). CD244^−/−^ mice were generated as previously described [17]. CD244^fl/fl^ mice were generated by Cyagen Biosciences (Santa Clara, CA, USA). CD244^fl/fl^ mice were bred with LysM-cre^+/+^ (B6.129P2-LysMtm1(cre)Ifo/J) mice from the Jackson Laboratory (Bar Harbor, ME, USA) to generate CD244^fl/fl^ and CD244^fl/fl^LysM-cre^+/−^ mice, and littermates were used. Female mice between 5 and 10 weeks of age were used. Mice were bred and maintained in a specific pathogen-free facility at Korea University.

### Cell lines and tumor models

B16F10 melanoma cells were purchased from American Type Culture Collection (ATCC). To establish subcutaneous tumors, 1 × 10^6^ B16F10 cells were injected into the right flank of mice, which formed a tumor with a 1-cm diameter within 1–3 weeks of injection. Tumors were measured regularly with digital calipers and tumor volumes were calculated by the formula: length × width × height / 2.

### Cell isolation

Single cell suspensions were prepared from the spleen and bone marrow (BM), followed by red blood cell removal using ammonium chloride lysis buffer. Monocytes were purified from the BM using the Monocyte Isolation Kit (Miltenyi Biotec, Auburn, CA, USA) according to the manufacturer’s instructions. Single cell suspensions from tumor tissues were prepared using the Mouse Tumor Dissociation Kit (Miltenyi Biotec, Auburn, CA, USA) and Percoll (GE Healthcare, Chicago, IL, USA) density gradient separation according to the manufacturer’s recommendations. For CD11b^+^ cell isolation, enriched single-cell suspensions from tumor lysates were labelled with biotinylated anti-CD11b antibody (BioLegend, San Diego, CA, USA), streptavidin-microbeads and separated on MACS columns (Miltenyi Biotec, Auburn, CA, USA).

### Bone marrow-derived macrophages (BMDM) and tumor-infiltrating monocyte differentiation

Enriched monocytes, whole BM cells, or magnetically isolated CD11b^+^ cells from B16F10 tumors were cultured in the presence of 50 ng/mL recombinant M-CSF (Peprotech, Cranbury, NJ, USA) in RPMI (Welgene, Gyeongsan-si, Korea) supplemented with 10% FBS, 10 mM HEPES, 20 µM 2-mercaptoethantol, 1% penicillin/streptomycin, and 1% non-essential amino acids.

### Mixed bone marrow monocyte transfer assay

Enriched monocytes from bone marrow of CD45.2 CD244^−/−^ mice and CD45.1 WT mice were mixed in 1:1 ratio and stained with CellTrace™ Far Red Cell Proliferation Kit (Thermo Fisher Scientific, Waltham, MA, USA). 2 × 10^6^ monocytes were injected intratumorally to tumor mass 14 days after B16F10 inoculation.

### BMDM and anti-PD-L1 adoptive transfer assays

Anti-PD-L1 antibody (200 ug, clone 10 F.9G2; Bio X Cell, West Lebanon, NH, USA) or rat IgG2b isotype control antibody (200 ug, clone LTF-2; Bio X Cell, West Lebanon, NH, USA) was injected intraperitoneally 2 times in 100 ul PBS on 7 and 10 days after inoculation of tumor cells. 5 × 10^5^ – 1 × 10^6^ of D + 7 differentiated WT or CD244^−/−^ BMDMs were injected twice by the intravenous route on the same day as the antibody injection.

### Flow cytometry

Typically, up to 1 × 10^6^ cells were incubated with Fc-block (2.4G2 clone; Bio X cell, West Lebanon, NH, USA) for 5 min at room temperature (RT) and surface staining was performed for 30 min at 4 °C in the dark. H-2Db gp100 tetramer-EGSRNQDWL-PE (MBL International, Woburn, MA, USA) staining was performed for 30 min at 4 °C in the dark, prior to surface staining. For intracellular staining, CD45^+^ enriched single cell suspensions of tumor or tumor-draining lymph nodes (TDLN) were incubated for 16 h with γ-irradiated B16F10 melanoma cells and brefeldin A. After incubation, cells were surface-stained, perforated and intracellularly stained with a BD fixation/permeabilization kit (BD Biosciences, San Jose, CA, USA) according to the manufacturer’s recommendations. Cells were run on a FACSCanto II flow cytometer (BD Biosciences, San Jose, CA, USA), and data were analyzed using FlowJo software (BD Biosciences, San Jose, CA, USA). A list of the antibodies used is provided in Supplementary Table 2.

### Quantitative PCR

Total RNA was extracted using TRIzol reagent (Thermo Fisher Scientific, Waltham, MA, USA). cDNA was synthesized using the TOPscript™ cDNA Synthesis Kit (Enzynomics, Daejeon, Korea). Real-time quantitative PCR was performed using SYBR Green (Bio-Rad, Hercules, CA, USA) on StepOnePlus™ (Applied Biosystems, Middlesex County, MA, USA). Gene expression was normalized to the expression level of GAPDH, and relative expression levels were calculated according to the 2^− ΔΔCt^ method. Genes were amplified using the primers listed in Supplementary Table 3.

### ELISPOT

After 3 days of BMDM differentiation, BMDMs and 5 × 10^4^ lymph node cells from OT-1 transgenic mice were co-cultured at various ratios with cognate OVA peptide (SIINFEKL; 1 µg/mL; Invivogen, San Diego, CA, USA). Cells were plated in the Mouse IFN-γ ELISPOTPLUS kit (ALP) (MABTECH, Nacka Strand, Sweden) and IFN-γ spots were detected after 48 h using ELISPOT reader systems (Autoimmun Diagnostika GmbH, Strassberg, Germany).

### Antigen presentation and phagocytosis assay

After 2 days of BMDM differentiation, 0.1-1 mg/mL of whole OVA protein (Invivogen, San Diego, CA, USA) or γ-irradiated and CFSE (Thermo Fisher Scientific, Waltham, MA, USA)-stained B16F10 melanoma cells were added to the culture. OVA peptide-conjugated MHC-I complex and MHC-II were labeled with anti-H-2 kb-SIINFEKL antibody and anti-MHC-II antibody (BioLegend, San Diego, CA, USA), respectively, to assess antigen presentation. CFSE fluorescence was measured to assess phagocytosis using a FACSCanto II flow cytometer (BD Biosciences, San Jose, CA, USA).

### Fluorescence microscopy

BMDMs cultured on confocal dishes were fixed and permeabilized with 4.2% paraformaldehyde solution and stained with anti-LC3B antibody (1:200) and Hoechst 33,342 (1:1000). After 2 h of incubation, the cells were washed and stained with Alexa555-rabbit IgG secondary antibody and incubated for 1 h at RT. All images were captured using the LSM700 confocal laser-scanning microscope (Carl Zeiss, Oberkochen, Germany) equipped with a 63× oil-immersive lens.

### scRNA-seq data analysis

A scRNA-seq dataset (accession ID: GSE121861; mouse syngeneic tumor, SCP398; human melanoma, SCP1162; human CRC, GSE127465; human NSCLC, GSE131928; human GBM) that was previously reported [10, 22–25] was obtained from the Single Cell Portal (https://singlecell.broadinstitute.org/single_cell) and Gene Expression Omnibus (GEO) (https://www.ncbi.nlm.nih.gov/geo/) database. From the SCP398 dataset, we collected unique molecular identifier (UMI) count matrix for cells selected based on the quality control criteria as well as the cell type annotations reported in the original study. Only the UMI count profiles for 1,391 cells corresponding to monocytes and macrophages, among the 16,291 cells in the dataset, were used for our analysis. The count matrix was normalized by cell-specific size factors using the Seurat (v4.0.6) R package [26] and subsequently log2-transformed after the addition of a pseudo-count of 1. Highly variable genes (HVGs) were identified using FindVariableFeatures in Seurat with a FDR < 0.05. Using these HVGs, we clustered 1,391 cells corresponding to monocytes and macrophages with the first 50 principal components (PCs) obtained for the HVGs using FindClusters function in Seurat. We then visualized the resulting subclusters of monocytes and macrophages using uniform manifold approximation and projection (UMAP) with the RunUMAP function in Seurat. Genes predominantly upregulated in each subcluster were identified using the FindAllMarkers function with an adjusted *P*-value of < 0.05 and log2-fold change of > 0.25. In addition, differentially expressed genes (DEGs) between CD244-positive and negative cells were identified using the same function. Functional enrichment analysis of DEGs was performed using ConsensusPathDB software (version 35) [27]. Gene ontology biological processes (GOBPs) and pathways enriched by the genes were identified as those with *P* < 0.05. Other scRNA-seq data were analyzed with similar methods described above.

### Bulk RNA-seq data analysis

We obtained two RNA-seq datasets of melanoma patients previously reported [28, 29]from the TCGA database (TCGA-SKCM; https://portal.gdc.cancer.gov) and GEO database (accession ID: GSE91061). We first estimated the proportions of CD244-positive and negative cells in individual melanoma tissue samples through cell deconvolution analysis using CIBERSORTx software with absolute mode and disabled quantile normalization [30]. The fragments per kilobase of transcript per million mapped reads (FPKM) values of individual melanoma tissue samples were uploaded to CIBERSORTx as a mixture file. For survival analysis, the patients were divided into two groups: with or without CD244-negative cell fractions. The cumulative event (death) rate was calculated for each patient group using the Kaplan–Meier method, and the survival curves of the two patient groups were compared using the Kaplan–Meier (log rank) test.

### Statistical analysis

All data are presented as mean ± standard error of the mean (S.E.M). Details on the sample size (number of samples) are indicated in the figure with dots. Number of repetitions and statistical tests are listed in the figure legends. Generally, Student’s *t*-test was used to determine the statistical significance between the two groups. One-way ANOVA test was used to determine the statistical significance between more than two groups. Two-way ANOVA test was used to determine significant differences between groups when more than one variable was being assessed. Significance was defined at *P* < 0.05. All analyses were performed using the Prism software (version 7.0; GraphPad Software, Inc., San Diego, CA, USA).

## Results

### Absence of CD244 signaling in monocyte-lineage cells suppresses melanoma tumorigenesis

**Fig. 1:**
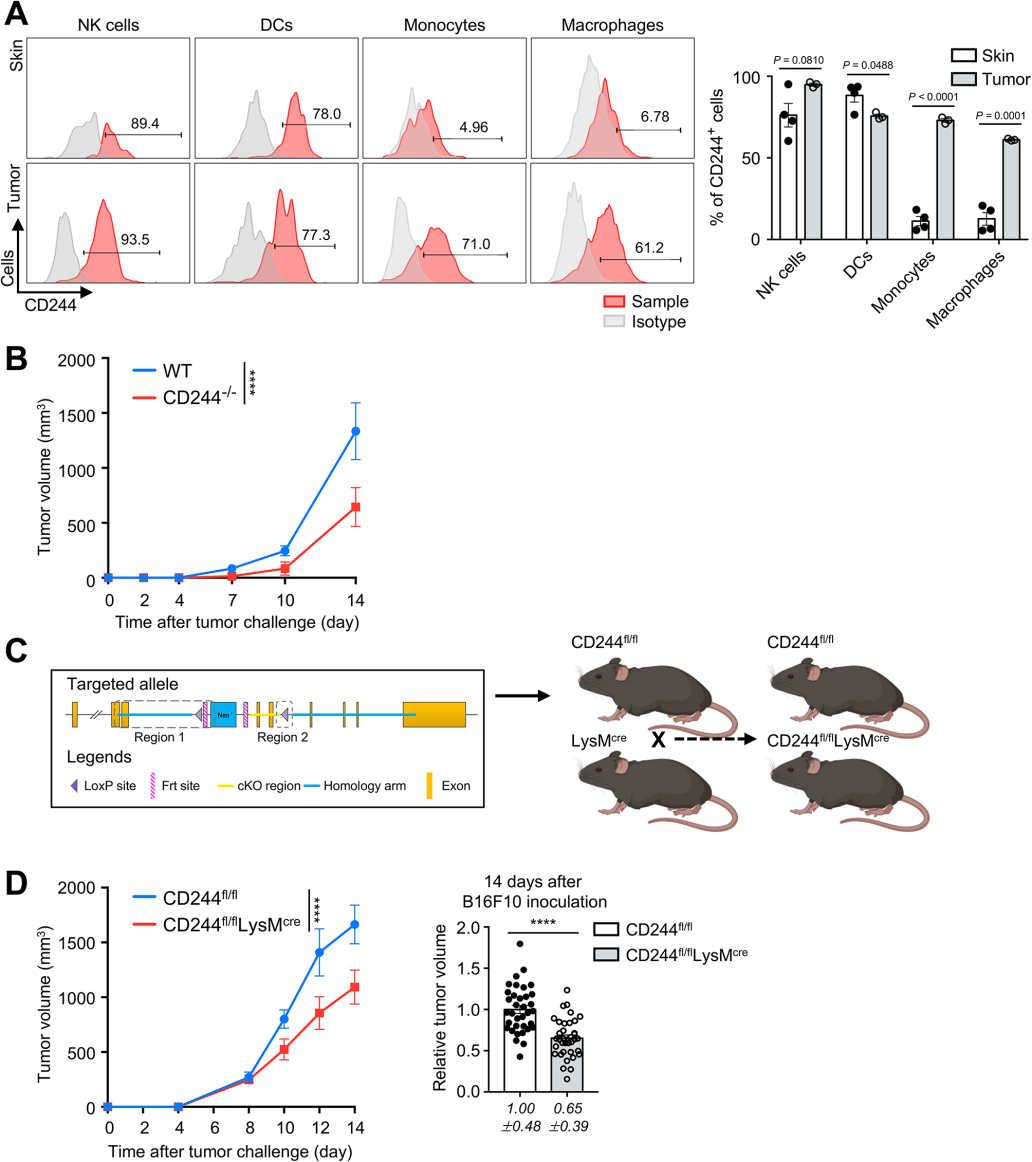
The lack of CD244 in monocytes inhibited melanoma tumorigenesis. **(A, B, D)** 1 × 10^6^ of B16F10 cells were subcutaneously inoculated into the right flank of mice. **(A)** CD244 expression in NK cells, DCs, monocytes and macrophages in the skin of WT naïve mice and within the B16F10 tumor mass of WT tumor-bearing mice, 14 days after tumor inoculation, was analyzed using flow cytometry. The skin samples were derived from mice that did not undergo tumor inoculation and obtained from the identical location as the tumor site in mice subjected to tumor inoculation. Representative histograms **(left)** display surface CD244 expression on NK cells, DCs, monocytes and macrophages from the skin **(top)** and the tumor **(bottom)**. The percentages of CD244-expressing cells in NK cells, DCs, monocytes and macrophages from both skin and tumor are presented as a bar graph **(right)**. **(B)** Tumor growth in WT and CD244^−/−^ mice is depicted (*n* = 4 in each group). **(C)** The process for generating littermate CD244^fl/fl^ and CD244^fl/fl^LysM^cre^ mice is illustrated. **(D)** B16F10 tumor growth in littermate CD244^fl/fl^ and CD244^fl/fl^LysM^cre^ mice is shown (*n* = 4 in each group) (**left**). The relative tumor volume on 14 days after tumor inoculation (n (number of samples) = 35 of CD244^fl/fl^ and 36 of CD244^fl/fl^LysM^cre^ mice) (**right**). Significance was indicated as ****P < 0.0001, and the statistical analysis was performed using two-way ANOVA **(B, D (left))** or unpaired Student’s *t*-test **(A, D (right))**. Data are representative of two **(A)**, three **(B)** or nine **(D (left))** or compiled from nine **(D (right))** independent experiments

To determine the impact of CD244 expression in tumor-infiltrating monocyte-lineage cells, we analyzed CD244 expression on monocytes and macrophages in the skin of C57BL/6 wild-type (WT) naïve mice and in tumor masses at 14 days following subcutaneous injection with 1 × 10^6^ B16F10 tumor cells. Our results showed that representative CD244-expressing cells, such as NK cells and DCs, had constitutive expression of CD244 in both skin and tumor tissues. However, monocytes and macrophages in the skin did not express CD244, while they significantly upregulated their CD244 expression after tumor inoculation (Fig. 1A). As shown in the right, the percentage of CD244-expressing cells increased up to 71.0% in the monocytes and 61.2% in the macrophage population within tumor. These findings suggest a tumor-specific role of CD244 in monocyte-lineage cells and imply its potential involvement in the immune response to tumors.

When compared the growth of B16F10 tumors in WT and CD244 whole knockout (CD244^−/−^) mice, we found that the growth of B16F10 tumors was significantly slower in CD244^−/−^ mice than in WT mice (Fig. 1B), consistent with previous results in a head and neck squamous cell carcinoma model [21]. The proportion of immune cell sub-populations in the spleen and tumor did not differ between WT and CD244^−/−^ mice (Supplementary Fig. 1A). Next, we examined whether a specific deletion of CD244 on monocyte-lineage cells within tumors could reduce tumor growth. To this end, we generated C57BL/6 background CD244^fl/fl^ mice and crossed them with LysM-cre^+/−^ mice to produce CD244^fl/fl^LysM^cre^ mice, in which CD244 was deleted specifically in the monocytes and macrophages (Fig. 1C and Supplementary Fig. 1B). Subsequently, we injected syngeneic B16F10 melanoma cells subcutaneously into these mice. Similar to the results obtained with CD244^−/−^ mice, monocyte-specific deletion of CD244 in CD244^fl/fl^LysM^cre^ mice significantly reduced tumor growth compared to littermate control CD244^fl/fl^ mice (Fig. 1D). Collectively, CD244 suppressed anti-tumor activity, and its expression was significantly increased in monocytes and macrophages within melanoma.

### Targeted CD244 deletion in monocyte-lineage cells enhances tumor antigen-specific CD8 T cell responses

**Fig. 2:**
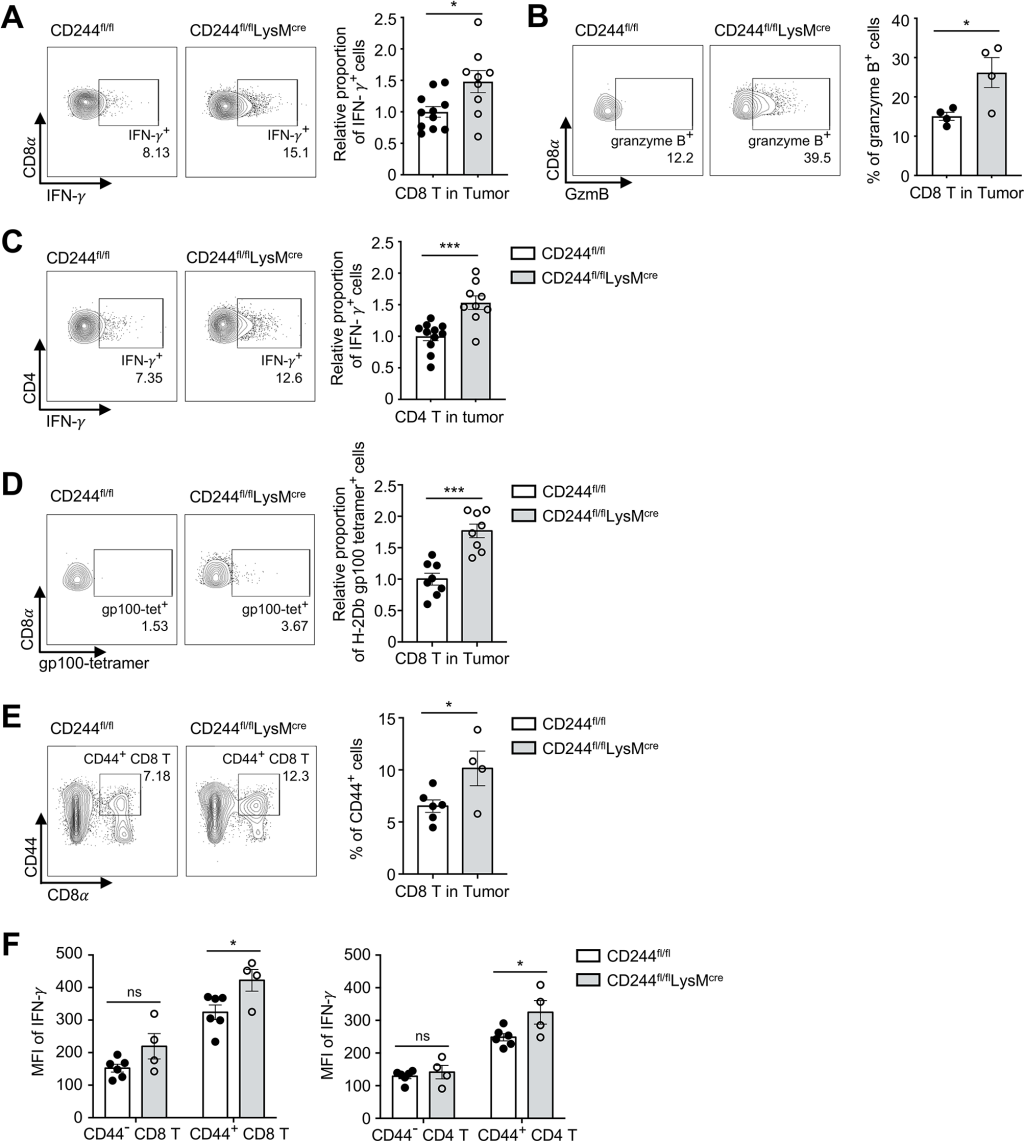
The deletion of CD244 on monocyte-lineage cells enhanced IFN-γ secretion from antigen-specific T cells. **(A-E)** The CD45^+^ cell population infiltrating the tumor was isolated using magnetic sorting and subjected to flow cytometry analysis 14 days after inoculating 1 × 10^6^ B16F10 cells into CD244^fl/fl^ and CD244^fl/fl^LysM^cre^ mice. IFN-γ and granzyme-B expression were measured after 16 h co-culture with γ-irradiated B16F10 cells. Expression of IFN-γ **(A)** and granzyme-B **(B)** in CD8 T cells, as well as IFN-γ expression in CD4 T cells **(C)**, gp100-specific TCR **(D)**, CD44 **(E)** expression in CD8 T cells and IFN-γ expression in CD44^−^ and CD44^+^ CD8 T cells **(left)** and CD4 T cells **(right) (F)** were shown. *P < 0.05; ***P < 0.001; unpaired Student’s *t*-test. Data are representative of two **(B, E, F)** or compiled from two **(A, C, D)** independent experiments

To delineate the mechanism underlying the observed accelerated tumor clearance in CD244^fl/fl^LysM^cre^ mice, we harvested tumor-infiltrating lymphocytes (TILs) from B16F10 tumor-bearing CD244^fl/fl^ and CD244^fl/fl^LysM^cre^ mice and quantified IFN-γ expression in response to γ-irradiated B16F10 tumor targets ex vivo. Although the proportion of tumor-infiltrating lymphocytes remained unchanged (Supplementary Fig. 2A), the expression of IFN-γ (Fig. 2A) and granzyme-B (Fig. 2B) in tumor-infiltrating CD8 T cells were significantly higher in CD244^fl/fl^LysM^cre^ mice compared to CD244^fl/fl^ mice. Moreover, both CD8 T cells in the tumor-draining lymph node (TDLN) (Supplementary Fig. 2B) and CD4 T cells in the tumor (Fig. 2C) and TDLN (Supplementary Fig. 2C), showed an increase in IFN-γ expression in CD244^fl/fl^LysM^cre^ mice compared to CD244^fl/fl^ mice.

To determine whether the increased IFN-γ and granzyme-B expression in CD8 T cells was associated with antigen-specific T cell responses, we examined the presence of melanoma-specific CD8 T cells with H-2Db gp100 tetramer. We found that the proportion of tetramer-positive CD8 T cells was significantly increased in the tumors of CD244^fl/fl^LysM^cre^ mice compared to CD244^fl/fl^ mice (Fig. 2D). Finally, by confirming increased CD44-expressing activated CD8 T cells [31] (Fig. 2E and Supplementary Fig. 2D) and IFN-γ expression in CD44-expressing T cells (Fig. 2F), we suggest that selective deletion of CD244 in monocyte-lineage cells enhances the antigen-specific activation of CD8 T cells.

### CD244 suppresses maturation of monocyte-lineage cells

**Fig. 3:**
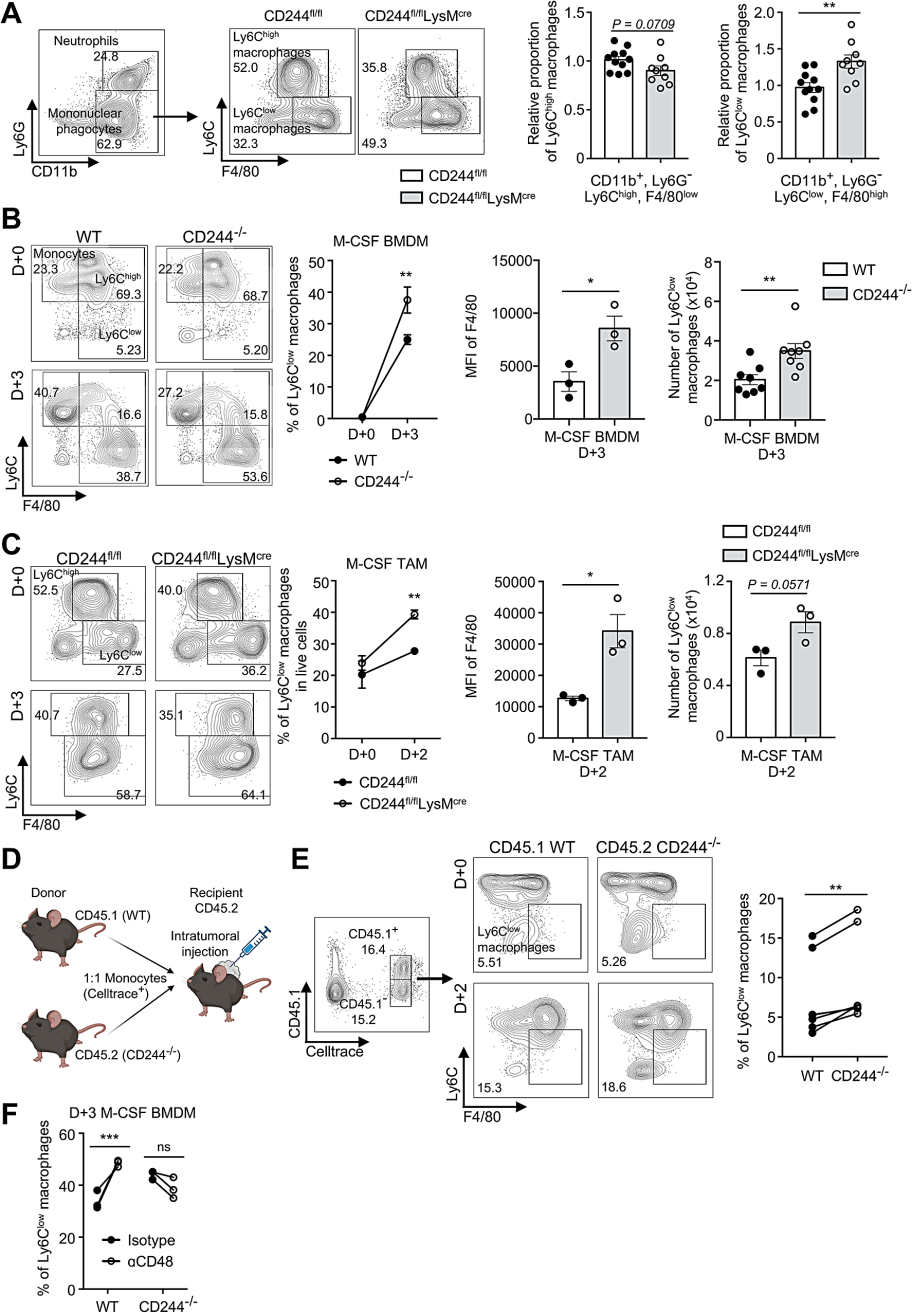
The lack of CD244 led to an increase in M1 macrophage populations. **(A)** After inoculating B16F10 cells into CD244^fl/fl^ and CD244^fl/fl^LysM^cre^ mice, the percentage of Ly6C^high^ macrophages (CD11b^+^Ly6G^−^Ly6C^high^F4/80^low^) and Ly6C^low^ macrophages (CD11b^+^Ly6G^−^Ly6C^low^F4/80^high^) within tumor was assessed 14 days later. Representative flow cytometry plots **(left)** and relative proportion of Ly6C^high^ macrophages **(middle)** and Ly6C^low^ macrophages **(right)** in CD45^+^ cells were shown. **(B-C)** Bone marrow cells **(B)** or tumor-infiltrating CD11b^+^ cells **(C)** were cultured with M-CSF for 3 days. Presented are a representative flow cytometry plot **(1st)**, proportion of Ly6C^low^ macrophages **(2nd)**, Mean fluorescence intensity (MFI) of F4/80 **(3rd)** and absolute Ly6C^low^ macrophage count **(4th)**. **(D-E)** Monocytes were isolated from bone marrow of CD45.1 WT and CD45.2 CD244^−/−^ mice, stained with CellTrace Far Red (CTFR), and mixed in a 1:1 ratio. This monocyte mixture was then injected directly into B16F10 tumor mass of WT CD45.2 recipient mice. Tumors were harvested after 48 h, and the ratio of CTFR^+^ CD45.1 and CD45.2 Ly6C^low^ macrophages was determined. **(D)** Schematic representation of in vivo differentiation experiment. **(E)** Demonstrated are a representative flow cytometry plots **(left)** and the proportion **(right)** of CTFR^+^ CD45.1 and CD45.2 Ly6C^low^ macrophages within tumors. **(F)** WT and CD244^−/−^ bone marrow cells were cultured for 72 h with M-CSF and treated either with an isotype control or an anti-CD48 antibody. The proportion of Ly6C^low^ macrophages was determined and presented. *P < 0.05; **P < 0.01; ***P < 0.001; ns, not significant; unpaired Student’s *t*-test **(A, B, C)** or two-way ANOVA **(B, C**) or paired Student’s *t*-test **(E, F)**. Data are representative of three **(B, C)** or two **(E, F)** or compiled from three **(A)** independent experiments

To assess the direct impact of CD244 deficiency in CD244^fl/fl^LysM^cre^ mice, we first analyzed the myeloid cell populations within the tumor mass and compared it to those in control CD244^fl/fl^ mice. We found no significant differences in the proportion of CD45^+^CD11b^+^ myeloid cells between the two groups. Additionally, neither the proportion of neutrophils (CD11b^+^Ly6G^+^) nor DCs (CD11b^+^Ly6C^−^F4/80^−^CD11c^+^MHCII^+^) was altered in CD244^fl/fl^LysM^cre^ mice (Supplementary Fig. 3A and B). However, the proportion of Ly6C^high^ macrophages (CD11b^+^Ly6G^−^Ly6C^high^F4/80^low^) was slightly decreased, while that of Ly6C^low^ macrophages (CD11b^+^Ly6G^−^Ly6C^low^F4/80^high^) was significantly increased in CD244^fl/fl^LysM^cre^ mice compared to CD244^fl/fl^ mice (Fig. 3A).

We found that the percentages of apoptotic Ly6C^high^ and Ly6C^low^ macrophages in the tumor were comparable between CD244^fl/fl^LysM^cre^ and CD244^fl/fl^ mice (Supplementary Fig. 3C). Subsequently, we investigated whether CD244 was involved in the regulation of macrophage maturation, given the decrease in Ly6C^high^ macrophages and increase in Ly6C^low^ macrophages in CD244^fl/fl^LysM^cre^ mice, while maintaining the percentage of total mononuclear phagocytes. Ly6C^high^CX3CR1^low^ monocytes originated from bone marrow rapidly differentiated into F4/80^+^ tumor-associated macrophages (TAMs) in tumor microenvironment and lose its Ly6C expression under stimulation by M-CSF [32]. In vitro macrophage differentiation assays using whole bone marrow (BM) cells in the presence of 50 ng/mL recombinant macrophage colony-stimulating factor (M-CSF) revealed a significant increase in both the proportion and the number of Ly6C^low^ macrophages in CD244^−/−^ bone marrow cultures (37.5% ± 0.030, 3.5 × 10^4^ ± 0.37 × 10^3^) in comparison to those in WT bone marrow cultures (25.0% ± 0.021, 2.0 × 10^4^ ± 0.26 × 10^3^) (Fig. 3B). Furthermore, ex vivo macrophage differentiation assays using tumor-infiltrating CD11b^+^ cells isolated from the tumors of CD244^fl/fl^LysM^cre^ mice resulted in an increased proportion and number of Ly6C^low^ macrophages, compared to those of CD244^fl/fl^ mice (Fig. 3C).

In the tumor microenvironment, various factors beyond M-CSF and GM-CSF have been shown to regulate macrophage maturation, including IL-3, IL-10, TGF-β, and physical factors such as extracellular matrix and hypoxia [33]. To directly confirm the role of CD244 in vivo, we performed adoptive transfer assay in which monocytes from the bone marrow of CD45.1 WT and CD45.2 CD244^−/−^ mice were transferred into tumor-bearing CD45.2 WT recipient mice using intratumoral injection (Fig. 3D). Similar to in vitro results, CD45.2 CD244^−/−^ monocytes differentiated more readily into Ly6C^low^ macrophages than WT monocytes within tumor mass in vivo (Fig. 3E).

To further investigate whether the increased macrophage population in CD244-deficient mice was associated with impaired signaling from the lack of CD244 and CD48 binding, we differentiated BM monocytes in the presence of monoclonal antibody (mAb) against CD48 to block its interactions with CD244. Anti-CD48 mAb treatment significantly increased the Ly6C^low^ macrophage population in the WT cultures, but not in the CD244^−/−^ cultures (Fig. 3F). Taken together, these findings suggest that the specific loss of CD244 signaling in tumor-infiltrating monocytes favors the differentiation of Ly6C^low^ macrophages.

### CD244 suppresses anti-tumorigenic functions of macrophages

**Fig. 4:**
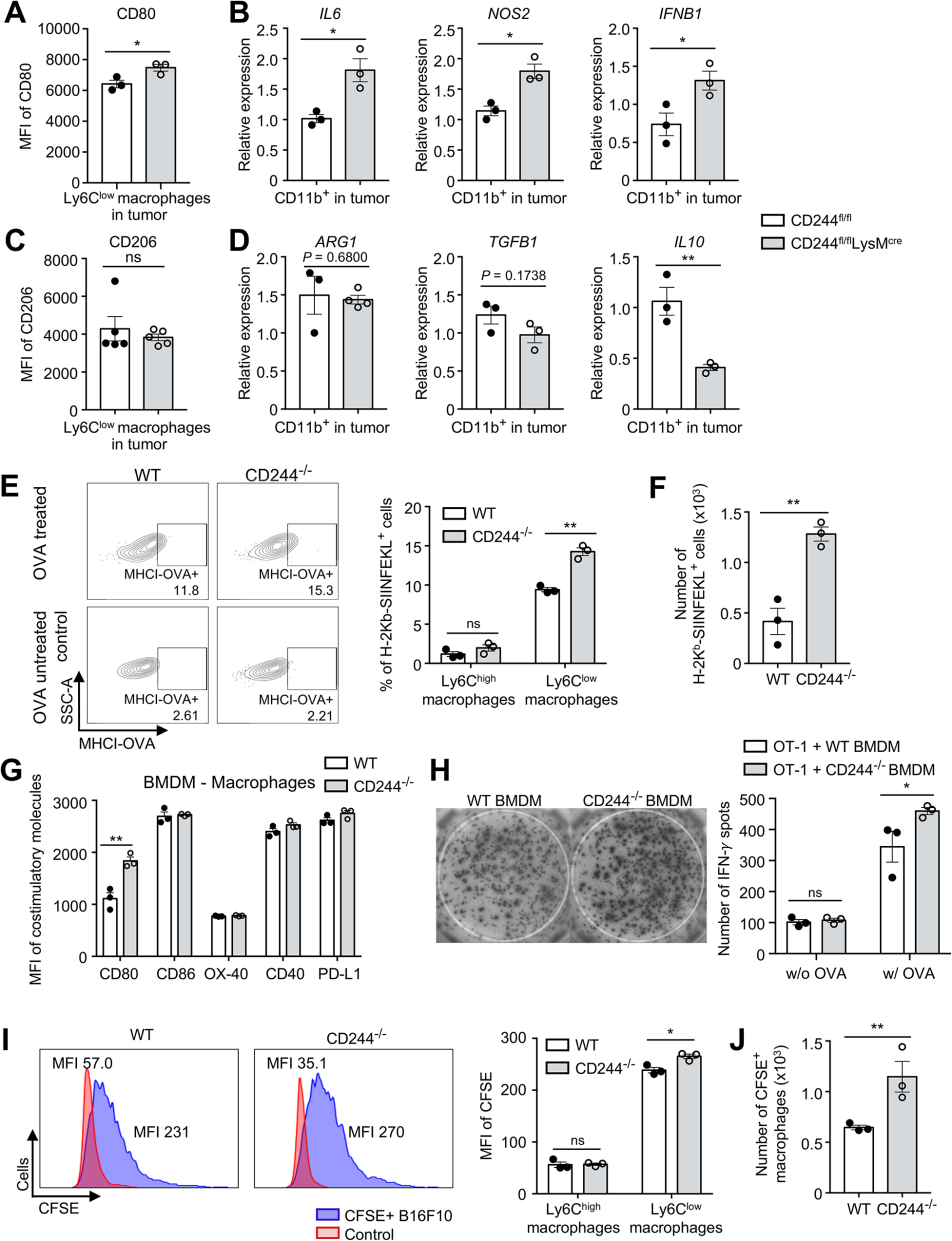
The deletion of CD244 on monocyte-lineage cells enhances antigen presentation and phagocytosis. **(A-D)** The population of tumor-infiltrating CD11b^+^ cells was isolated using magnetic sorting, and the expression of anti/pro-tumorigenic macrophage markers was evaluated using flow cytometry and real-time quantitative PCR. **(A)** MFI of CD80 in macrophages. **(B)** Relative mRNA expression level of anti-tumorigenic macrophage markers (*IL6, NOS2, IFNB1*). **(C)** MFI of CD206 in macrophages. **(D)** Relative mRNA expression level of pro-tumorigenic macrophage markers (*ARG1, TGFB1, IL10*). **(E-F)** Bone marrow cells were cultured with M-CSF and whole OVA protein. OVA presentation via MHC-I was measured using anti-H-2 kb-SIINFEKL antibody on day 3. Demonstrated are representative flow cytometry plots **(left)**, proportion **(right) (E)** and absolute number **(F)** of MHC-I-OVA complex expressing cells in WT and CD244^−/−^ BMDMs. **(G)** MFI of costimulatory molecules (CD80, CD86, OX-40, CD40, and PD-L1) on WT and CD244^−/−^ BMDM on day 3. **(H)** Differentiated BMDMs and OT-1 cells were co-cultured for 48 h with whole OVA protein. Representative photographs **(left)** and the number of IFN-γ spots **(right)** were counted for OT-1 cells co-cultured with either WT or CD244^−/−^ BMDMs. (**I-J**) Differentiated BMDMs were co-cultured with CFSE-stained B16F10 cells, and phagocytic activity was assessed 24 h later by measuring CFSE fluorescence in macrophages. Presented are representative flow cytometry plots **(left)** and MFI of CFSE (**right**) in monocytes and macrophages **(I)** and the absolute number of CFSE^+^ macrophages **(J)** from WT and CD244^−/−^ BMDMs. *P < 0.05; **P < 0.01; ns, not significant; unpaired Student’s *t*-test. Data are representative of two **(A-D, G, H)** or three **(E, F, I, J)** independent experiments

We next investigated the mechanism underlying increased function of CD8 T cells in CD244^fl/fl^LysM^cre^ mice. We analyzed tumor samples from CD244^fl/fl^ and CD244^fl/fl^LysM^cre^ mice using flow cytometry and real-time quantitative PCR to confirm whether CD244 regulates the function of macrophages. We found that the increased number of Ly6C^low^ macrophages in CD244^fl/fl^LysM^cre^ mice was accompanied by a significant upregulation of anti-tumorigenic macrophage markers and cytokines, including CD80 (Fig. 4A), *NOS2*, *IL6*, and *IFNB1* (Fig. 4B), in CD11b^+^ sorted tumor-infiltrating macrophage populations. Although there was no difference in the expression of markers representing pro-tumorigenic macrophages, such as CD206 (Fig. 4C), *ARG1*, and *TGFB1*, the expression of *IL-10* was significantly reduced in CD244^fl/fl^LysM^cre^ macrophages (Fig. 4D).

Next, we examined whether CD244 deletion could have influenced MHC class I-mediated antigen presentation. We incubated whole OVA proteins with WT and CD244^−/−^ BMDM cultures for 24 h after differentiating BM for 2 days. Our flow cytometry analysis using anti-H-2kb-SIINFEKL mAb revealed that CD244^−/−^ Ly6C^low^ macrophages exhibited increased surface OVA presentation compared to WT Ly6C^low^ macrophages (left panel, Fig. 4E). In contrast, Ly6C^high^ macrophages showed poor antigen presentation, with no discernible difference between WT and CD244^−/−^ (right panel, Fig. 4E). We found no significant change in the level of MHC class II surface expression (Supplementary Fig. 3D); however, increased macrophage differentiation in CD244^−/−^ BMDM culture further increased the number of macrophages expressing MHC-I-OVA complex (Fig. 4F) and MHC-II molecules (Supplementary Fig. 3E) than the WT BMDM culture. Furthermore, similar to in vivo conditions, the expression of CD80 costimulatory molecules essential for antigen presentation in BMDM was increased, while that of CD86, OX-40, CD40, and PD-L1 remained unchanged (Fig. 4G). To directly confirm the role of CD244 on macrophages in antigen-specific immune responses, we isolated CD8 T cells from the lymph nodes of OT-1 transgenic mice and co-cultured them with differentiated WT or CD244^−/−^ BMDM and stimulated with ovalbumin protein. Consistent with our hypothesis, OT-1 cells secreted significantly more IFN-γ when co-cultured with CD244^−/−^ BMDM than when co-cultured with WT control (Fig. 4H). These results directly support that CD244-deficiency in monocyte-lineage cells enhances the antigen-specific CD8 T cell response.

To determine whether phagocytosis against solid cancer is also regulated by CD244, we co-cultured BMDMs with carboxyfluorescein succinimidyl ester (CFSE) stained B16F10 cells. The proportion of CFSE^+^ macrophages were determined via flow cytometry as a measure of direct phagocytosis. As seen in Fig. 4I, CD244^−/−^ Ly6C^low^ macrophages exhibited significantly higher phagocytic activity (Fig. 4I and Supplementary Fig. 3F) than WT. Also, similar to antigen presentation, Ly6C^low^ macrophages in CD244^−/−^ BMDM further increased the number of CFSE^+^ macrophages undergoing phagocytosis (Fig. 4J). In parallel experiments, it seems that CD244 does not contribute to antigen presentation and phagocytosis in neutrophils and DCs. Consequently, the deletion of CD244 in these cells does not impact antigen-specific CD8 T cell activation. (Supplementary Fig. 3G-H). Collectively, these data demonstrate that CD244 on monocyte-lineage cells promotes tumor growth by inhibiting antigen presentation and phagocytotic functions of macrophages.

### ER stress increases the expression of CD244 on immunosuppressive monocytes, which ultimately inhibits autophagy

**Fig. 5:**
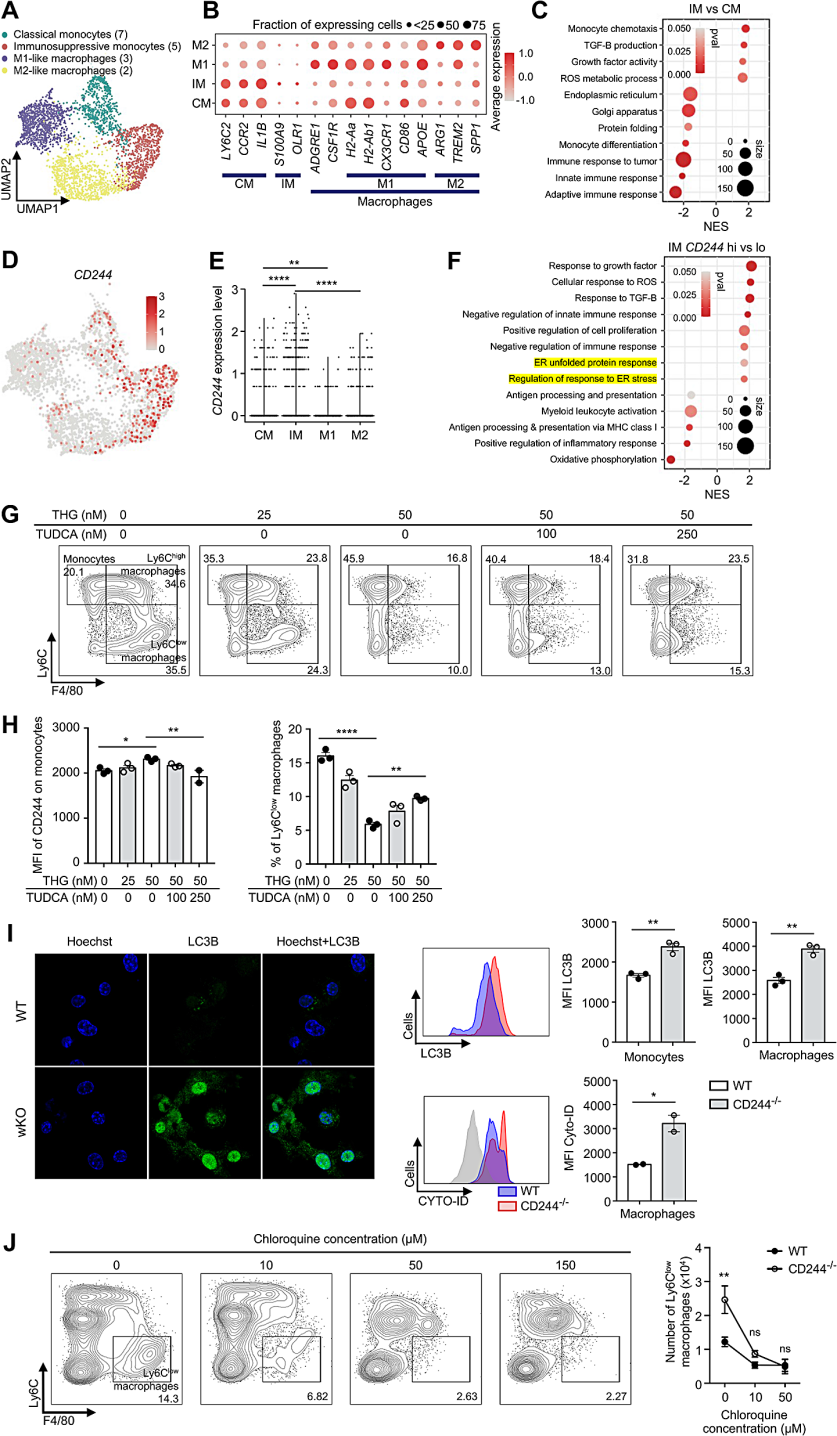
The expression of CD244 is increased in response to endoplasmic reticulum (ER) stress in immature monocytes. **(A-F)** The single cell RNA sequencing (scRNA-seq) data (GSE121861) was downloaded and re-analyzed. GSE121861 contained scRNA-seq data of 6 syngeneic mice tumor model (CT-26, EMT-6 : BALB/C; MC-38, LL2, B16F10 : C57B6/J; Sa1N : A/J). Each mouse tumor was harvested when it reached 100–200 mm. **(A)** Uniform manifold approximation and projection (UMAP) plot showing 4 clusters of myeloid cells among total 16 clusters containing 3 lymphoid [11, 13, 15] and 4 myeloid [2, 3, 5, 7] clusters, cancer associated fibroblasts (CAF), and tumor cells (CT26, LL2, MC-38, Sa1N, B16F10 and EMT-6). **(B)** The dotplot illustrated markers for classical monocytes (CM), immunosuppressive monocytes (IM), M1-like macrophages (M1), and M2-like macrophages (M2). **(C)** Gene set enrichment analysis (GSEA) result predicted from differentially expressed genes (DEGs) of IM compared to CM. **(D)** The UMAP plot depicted CD244 expression in 4 monocyte-lineage cell clusters. **(E)** A graph presented CD244 expression levels on CM, IM, M1, and M2. **(F)** GSEA result was predicted from DEGs of CD244-high IMs compared to CD244-low IMs. **(G-H)** Thapsigargin (THG) and Taurosodeoxycholic acid (TUDCA), an inducer and an inhibitor of ER stress, were administered to BMDMs along with M-CSF, and the cells were cultured for 3 days. **(G)** Representative flow cytometry plots displayed the monocyte and macrophage populations. **(H)** MFI of CD244 in monocytes **(left)** and the proportion of macrophages **(right)** were evaluated after treatment with THG alone or co-treatment with THG and TUDCA. **(I)** WT and CD244^−/−^ BMDMs were stained with Hoechst 33,342 and rabbit anti-mouse LC3B antibody, followed by a secondary anti-rabbit IgG-AlexaFlour555 antibody. LC3B expression on BMDMs was assessed using Immunofluorescence **(left)** and flow cytometry **(right; top)**. Autophagosome formation in WT and CD244^−/−^ BMDMs was measured by Cyto-ID staining **(right; bottom)**. **(J)** BMDMs were treated with chloroquine, an inhibitor of autophagolysosome formation, and M-CSF and cultured for 3 days. Representative flow cytometry plots demonstrated changes in the monocyte/macrophage population **(left)** and the number of macrophages **(right)**. *P < 0.05; **P < 0.01; ***P < 0.001; ****P < 0.0001; one-way ANOVA **(E, H**) or unpaired Student’s *t*-test **(I)** or two-way ANOVA **(J)**. The data represent two **(G-J)** independent experiments

To delineate the molecular pathways downstream of CD244 in monocyte-lineage cells, we analyzed the previously reported mouse syngeneic tumor single-cell RNA sequencing (scRNA-seq) dataset (GSE121861) [22]. Among the 7 CD45 (*PTPRC*)^+^ immune cell clusters (Supplementary Fig. 4A and B), we identified 4 clusters of monocyte-lineage cells with an expression pattern of known markers, such as those of classical monocytes (CM; *LY6C2, CCR2, IL1B*), immunosuppressive monocytes (IM; *S100A9, OLR1*), macrophages (*ADGRE1, CSF1R*), M1-like macrophages (M1; *H2-Aa, H2Ab1, CX3CR1, CD86, APOE*), and M2-like macrophages (M2; *ARG1, TREM2, SPP1*) (Fig. 5A and B). As previously reported, IMs exhibited significant upregulation of genes related to TGF-β production. On the other hand, monocyte differentiation, immune response, protein folding, Golgi apparatus, and endoplasmic reticulum (ER)-related genes showed substantial downregulation in IMs when compared to CMs [34]. (Fig. 5C). The expression of CD244 in monocyte-lineage cells was enriched on IMs compared to CMs and M1/M2-like macrophages (Fig. 5D and E). We also examined other members of SLAMF receptors, but only CD244 (SLAMF4) was intensively expressed on IMs (Supplementary Fig. 4C).

To elucidate the molecular pathways of CD244 in macrophages, we divided the IM population into two groups based on their CD244 expression level and predicted enriched pathways through their differentially expressed genes (DEGs). Consistent with our mouse data, we observed that IMs with low CD244 expression exhibited an elevated expression of genes associated with inflammatory response and MHC-I mediated antigen presentation, while CD244 high IMs showed an upregulation of pathways associated with proliferation, negative regulation of immune response, and ER stress responses which are major driver of immunosuppressive monocytes (Fig. 5F and Supplementary Fig. 4D).

Based on scRNA-seq data analysis, we hypothesized that upregulation of CD244 could be associated with ER stress in monocytes. Indeed, when we treated WT BMDM cultures with the ER stress inducer thapsigargin (THG), the surface expression of CD244 was increased on monocytes while the proportion of Ly6C^low^ macrophages decreased. Treatment with the ER stress inhibitor tauroursodeoxycholic acid (TUDCA) reversed the upregulation of CD244 expression by THG and restored the decreased Ly6C^low^ macrophage population (Fig. 5G and H). The addition of anti-CD48 antibody to THG-treated BMDMs led to a slight increase in Ly6C^low^ macrophages (Supplementary Fig. 4E). Together, our data support the hypothesis that CD244 is preferentially expressed on immunosuppressive monocytes and that its expression is increased in response to ER stress.

To investigate the signaling pathways initiated from CD244 on monocyte-lineage cells, we first checked the expression of classical adaptor molecules of CD244 using scRNA-seq dataset. Surprisingly, our analysis revealed that the activating adaptor molecule, *Sh2d1a* (SAP), is expressed only in T and NK cells, but not on monocyte-lineage cells. Expression of inhibitory molecules, such as EAT-2, ERT and SHP-2 were comparable in T/NK and monocyte-lineage cells. Recently, it was shown that Beclin-1 and Vps-34, which comprise the autophagy initiation complex, can be sequestered by the signaling domain of CD244 [35]. We found that expression of Beclin-1 was significantly elevated in monocyte-lineage cells rather than lymphocyte populations (Supplementary Fig. 5A). Since autophagy is a critical process for macrophage function and differentiation [36–38], we hypothesized that upregulation of CD244 expression induced by ER stress could potentially impede the differentiation and function of macrophages via autophagy regulation. Indeed, M-CSF-treated CD244^−/−^ BM cells differentiated into monocytes/macrophages had increased LC3 lipidation and autophagosome formation compared to WT cells (Fig. 5I). Furthermore, when BMDM cultures were treated with autophagy inhibitors, chloroquine, the fusion of autophagosomes were blocked and significantly reduced Ly6C^low^ macrophage differentiation was observed, especially in CD244^−/−^ cells compared to WT (Fig. 5J). These results suggest that the increased formation of autophagosomes in CD244^−/−^ monocytes/macrophages may have facilitated macrophage differentiation. Moreover, treatment with the PI3KC3 (Vps-34) inhibitor Ly294002 reduced macrophage differentiation in both WT and CD244^−/−^ cells (Supplementary Fig. 5B), with LC3B expression in CD244^−/−^ macrophages similarly reverting to WT levels as Ly294002 concentration increased (Supplementary Fig. 5C). These results indicate that increased Vps-34 availability in CD244^−/−^ monocytes/macrophages increased LC3B cleavage and autophagosome formation, ultimately leading to an increased macrophage differentiation. Overall, these data demonstrate a previously unidentified association of ER stress, CD244 upregulation, autophagy, and macrophage differentiation.

### CD244 deficient macrophages increase memory T cells and potentiates anti-PD-L1 therapy

**Fig. 6:**
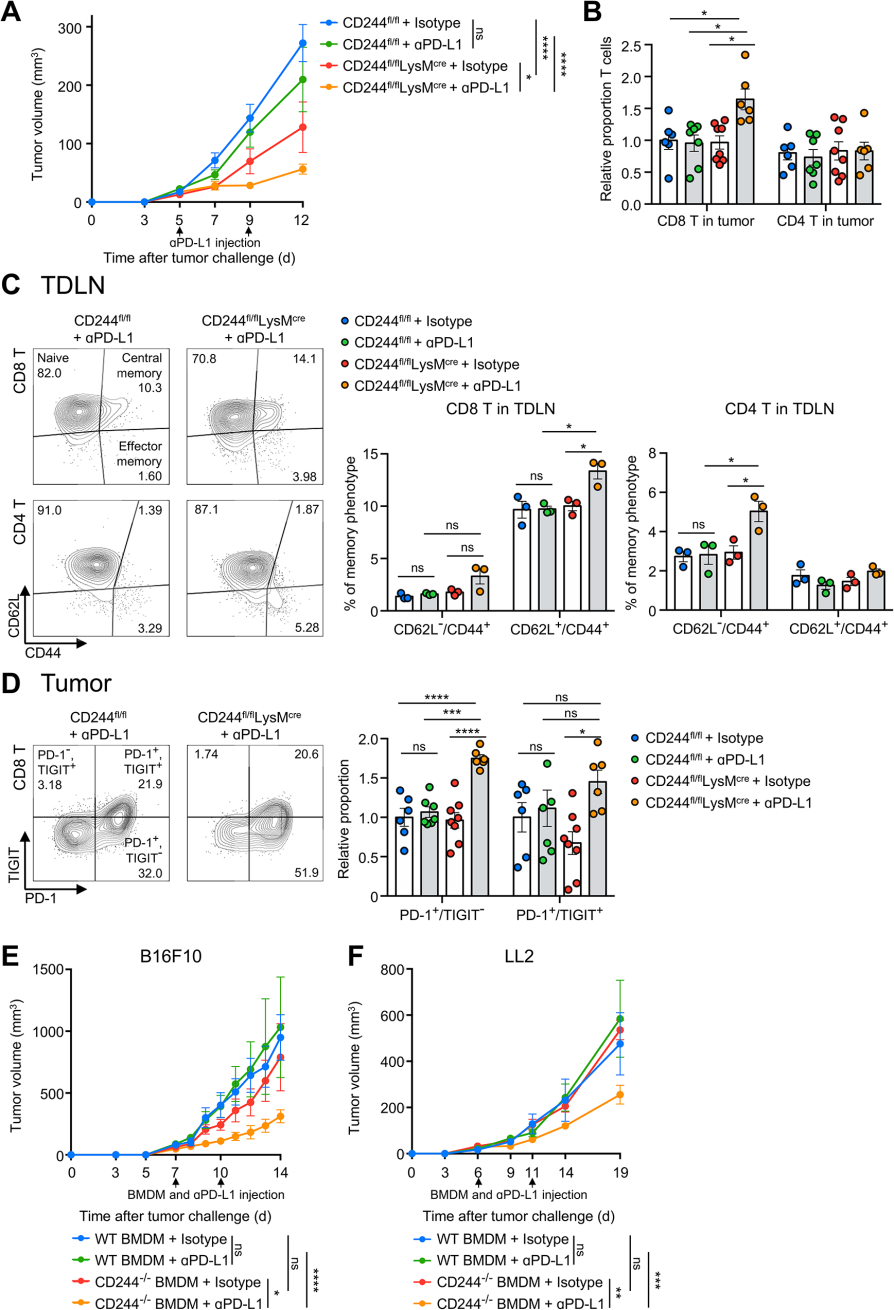
Macrophages lacking CD244 significantly delay tumor growth by increasing memory T cell populations when combined with anti-PD-L1 antibody. (**A to D**) CD244^fl/fl^ and CD244^fl/fl^LysM^cre^ mice were administered either anti-PD-L1 antibody or the corresponding isotype antibody on 5 and 9 days following B16F10 injection. Analysis of CD8 and CD4 T cells from both the tumor and tumor-draining lymph nodes (TDLN) was conducted 12 days after tumor inoculation. **(A)** A graph of B16F10 tumor growth. **(B)** Relative proportion of CD8 and CD4 T cells within the tumor. **(C)** Illustrated are a representative flow cytometry plots **(left)**, proportion of effector memory (CD62L^−^CD44^+^) and central memory (CD62L^+^CD44^+^) CD8 **(middle)** and CD4 **(right)** T cells in the TDLN. **(D)** Representative flow cytometry plots **(left)**, proportion of PD-1^+^TIGIT^−^ and PD-1^+^TIGIT^+^ cells among total CD8 T cells in the tumor. **(E, F)** After tumor inoculation, WT mice were co-administered twice with either WT or CD244^−/−^ BMDMs, along with either isotype antibody or anti-PD-L1 antibody. The growth of B16F10 **(E)** and LL2 **(F)** tumors was observed and evaluated in these mice. *P < 0.05; **P < 0.01; ***P < 0.001; ****P < 0.0001; ns, not significant; two-way ANOVA **(A, E, F)** or one-way ANOVA **(B-D)**. The data represents two **(A, C, E, F)** and compiled from two **(B, D)** independent experiments

Our data reveal that CD244 suppresses anti-tumor immunity by decreasing monocytes differentiation through autophagy regulation as well as phagocytic and antigen-presenting functions of macrophages, leading to an attenuated adaptive immune response. These findings suggest that macrophages lacking CD244 could serve as a therapeutic modality to enhance the efficacy of T cell-dependent immunotherapies. To test this hypothesis, we treated CD244^fl/fl^LysM^cre^ mice challenged with B16 melanoma with anti-PD-L1 antibody and compared the tumor growth with control CD244^fl/fl^ mice (Fig. 6A). Consistent with the previous findings that B16F10 tumors are largely refractory to PD-1/PD-L1 blockade therapy [39, 40], anti-PD-L1 antibody treatment in B16F10 tumor-bearing CD244^fl/fl^ mice did not induce significant changes in tumor growth. However, anti-PD-L1 antibody treatment further decreased the size of tumors in CD244^fl/fl^LysM^cre^ mice compared to the isotype antibody treatment (Fig. 6A). Interestingly, the proportion of CD8 T cells significantly increased after anti-PD-L1 antibody treatment, but this effect was observed exclusively in CD244^fl/fl^LysM^cre^ mice (Fig. 6B). Along with increased CD8 T cell population, central memory (CD62L^+^CD44^+^) CD8 T cells and effector memory (CD62L^−^CD44^+^) CD4 T cells were increased in TDLN of CD244^fl/fl^LysM^cre^ mice receiving the anti PD-L1 antibody (Fig. 6C and Supplementary Fig. 6A). In addition, the proportion of PD-1^+^ and TIGIT^−^ CD8 T cells was significantly increased only in the tumors of CD244^fl/fl^LysM^cre^ mice receiving the anti-PD-L1 antibody, whereas the proportion of severely exhausted PD-1 and TIGIT double-positive CD8 T cells [4] did not exhibit any significant changes compared to CD244^fl/fl^ (Fig. 6D).

To assess the therapeutic potential of CD244-deficient macrophages in the preclinical setting, we performed adoptive transfer of CD244^−/−^ BMDM in the presence of an anti-PD-L1 antibody to B16 melanoma-bearing WT mice. While the adoptive transfer of CD244^−/−^ BMDM into B16F10 tumor-bearing mice had a modest effect in suppressing tumor growth, the combination of CD244^−/−^ BMDM and anti-PD-L1 antibody resulted in significant reduction in the growth of B16F10 tumors (Fig. 6E). Other cold tumor model, Lewis lung carcinoma (LL2) also showed similar results (Fig. 6F). These data suggest that targeting CD244 on monocytes/macrophages potentially converts exhausted T cells into memory phenotypes and sensitize PD-L1 blockade in melanoma.

#### CD244 expression on monocytes/macrophages is negatively correlated with patient survival

**Fig. 7:**
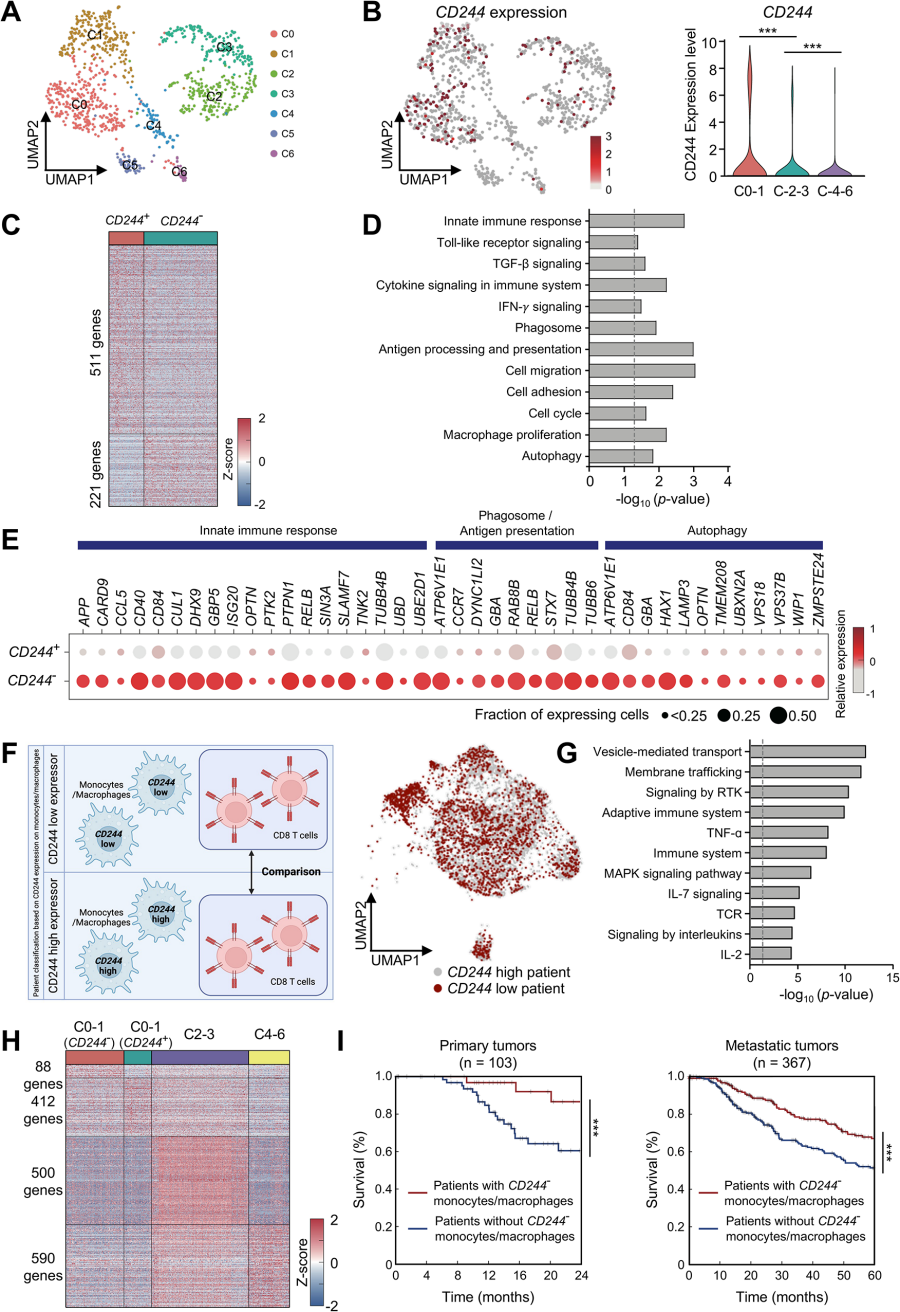
Single-cell RNA sequencing (scRNA-seq) data combined with deconvolution analysis unveil that CD244 plays a role in determining the destiny of monocytes/macrophages differentiation and influences melanoma patient survival. The scRNA-seq data of 16,291 immune cells from 48 tumor samples of melanoma patients treated with checkpoint inhibitors (GSE120575) was downloaded and subjected to re-analysis. **(A)** UMAP plot showing seven subclusters (C0–6) of monocytes/macrophages identified from scRNA-seq data. **(B)** UMAP plot showing CD244-expressing monocytes/macrophages **(left)** and CD244 expression levels in three cell groups (C0–1, C2–3 and C4–6) **(right)**. **(C)** Heatmap showing DEGs between CD244^+^ and CD244^−^ monocytes/macrophages in C0–1. **(D)** Cellular pathways enriched by 221 genes upregulated in CD244^−^ monocytes/macrophages. **(E)** The expression of genes preferentially involved in the innate immune response, phagosome/antigen presentation, and autophagy was assessed in CD244^+^ and CD244^−^ monocytes/macrophages **(F)** A schematic representation is provided to illustrate the classification of patients based on the expression level of CD244 in monocytes/macrophages **(left)**. The UMAP plot demonstrates the distribution of CD8 T cells among patients classified as CD244 low and high, based on the expression levels of monocytes/macrophages **(right)**. **(G)** Cellular pathways enriched by the genes upregulated in CD8 T cells of CD244 low patients (based on monocytes/macrophages); presented as –log_10_ (*P*-value). **(H)** Genes predominantly upregulated in C2–3, C4–6, CD244^+^ and CD244^−^ monocytes/macrophages in the C0–1. **(I)** DEGs in C2–3, C4–6 and CD244^+^ and CD244^−^ within the C0–1 were deconvoluted to TCGA-SKCM bulk RNA-seq data. The estimated survival of patients was analyzed based on the presence or absence of CD244-negative monocytes/macrophages. The overall survival of patients with primary tumors **(left)** or metastatic tumors **(right)** was assessed. ***P < 0.001; one-way ANOVA **(B)** or Kaplan–Meier (log rank) test **(G)**

To determine the clinical significance of CD244 as an immune checkpoint receptor on monocytes/macrophages within the TME, we re-analyzed a previously reported single-cell RNA-seq dataset (SCP398 from Single Cell Portal) of CD45^+^ immune cells isolated from human melanoma tissues [10]. Among the 16,291 cells reported in the original study, we focused on 1,391 cells annotated as monocytes and macrophages. To identify the CD244-negative monocytes/macrophages in these cells, we further clustered them into seven subclusters (C0–6, Fig. 7A) using the shared nearest neighbor clustering method in Seurat. CD244 expression was categorized into the following three groups (Fig. 7B): (1) C0–1 with relatively high enrichment of CD244 expression, (2) C2–3 with low enrichment of CD244 expression, and (3) C4–6 with no expression of CD244. Of these groups, we focused on C0–1, with significant numbers of both CD244-positive and CD244-negative monocytes/macrophages, for a fair comparison between the two cell types. A total of 511 and 221 genes were identified as predominantly upregulated in CD244-positive and CD244-negative monocytes/macrophages, respectively (Fig. 7C). The 221 genes predominantly expressed in CD244-negative monocytes/macrophages were primarily associated with phagocytosis (phagosome), antigen processing and presentation, and autophagy (Fig. 7D and E), which is consistent with the results found in CD244^fl/fl^LysM^cre^ mice challenged with B16F10 melanoma. Next, we examined whether CD244 low monocytes/macrophages could enhance antigen-specific T cell activity in humans, similar to what we observed in our mouse model. We classified patients into CD244 high and CD244 low groups according to their average expression level of CD244 on G3 monocytes/macrophages and compared their CD8 T cells, regardless of CD244 expression in T cells. (Fig. 7F and Supplementary Table 1). We obtained 207 genes predominantly upregulated in CD8 T cells from CD244 low patients and these DEGs were primarily associated with cytokine signaling, TCR signaling and the adaptive immune system (Fig. 7G).

The presence of CD244-negative monocytes/macrophages and their impact on T cell activity in human melanoma tissues prompted us to evaluate the clinical implications of CD244 on monocyte-lineage cells in the pathogenesis of human melanoma. To this end, we obtained bulk RNA-seq datasets generated from the tumor tissues of 470 melanoma patients (103 primary and 367 metastatic tumors) [28] from The Cancer Genome Atlas (TCGA) database (TCGA-SKCM). We then identified genes that were predominantly upregulated in the following four cell types identified from the two bulk RNA-seq dataset analyses: 1 & 2) CD244-negative and CD244-positive cells in C0–1, 3) cells in C2–3, and 4) cells in C4–6 (Fig. 7H). Notably, 511 and 221 genes identified from C0–1 only (Fig. 7C) were reduced to 412 and 88 genes, respectively, when we further filtered out the genes exhibiting increased expression in cells in C2–3 or C4–6 to ensure the specificity of their expression in C0–1 compared to C2–6. The proportions of the four cell types in individual tumor tissues from the TCGA dataset were estimated using CIBERSORTx [30], with the genes upregulated in each cell type. We then compared the survival of patients with and without CD244-negative monocytes/macrophages and found that patients with CD244-negative monocytes/macrophages showed better overall survival than those without CD244-negative monocytes/macrophages in two different patient cohorts with primary (2-year survival; Fig. 7I, left) and metastatic (5-year survival; Fig. 7I, right) tumors. These data are consistent with our results showing delayed tumor growth in monocyte/macrophage-specific CD244-deficient mice (Fig. 1B and Supplementary Fig. 7A and B).

Lastly, we examined if our results on CD244 on monocyte-lineage cells can be expanded to other tumor models beyond melanoma. We obtained 3 different scRNA-seq datasets of colorectal cancer (SCP1162), non-small cell lung cancer (GSE127465), and glioblastoma (GSE131928) patients. Pathway enrichment analysis on DEGs of CD244-negative monocytes/macrophages showed results similar to Fig. 7D, suggesting the genes were associated with the innate immune system, phagocytosis, antigen presentation, and autophagy (Supplementary Fig. 7C, D and E). Together, these data suggest that CD244 functions as a novel immune checkpoint receptor that restricts function and differentiation of monocytes/macrophages in cancer patients.

## Discussion

While the role of CD244 has been extensively studied in lymphoid cells, its function in myeloid cells is still not fully understood. In this study, we provide direct evidence using CD244^fl/fl^LysM^cre^ conditional knockout and CD244 whole knockout mice, that CD244 serves as a pivotal immune checkpoint receptor, impeding anti-tumor immunity within myeloid cells. Flow cytometry and RNA sequencing data demonstrated a significant upregulation of CD244 expression in response to ER stress within the tumor mass. The heightened expression of CD244 led to the subsequent inhibition of the autophagy pathway in monocytes. This inhibition directly impeded MHC-I-mediated antigen presentation, hindering the generation of Ly6C^low^ macrophages with anti-tumorigenic properties. The reduction in anti-tumorigenic macrophages had a cascading effect, impairing both phagocytosis and MHC-II-mediated antigen presentation within the overall monocyte-lineage cell clusters. Furthermore, transcriptome analysis of human melanoma patients revealed a potential prognostic significance of CD244-negative monocytes/macrophages in both primary and metastatic tumors. The increased proportion of CD244-negative monocytes/macrophages correlated with improved patient survival, suggesting that CD244-negative monocytes/macrophages could serve as a biomarker in these cases. Moreover, in B16 melanoma and Lewis lung carcinoma (LL2)-bearing mice, the synergistic application of anti-PD-L1 antibody with CD244-deficient macrophages significantly enhanced tumor rejection, surpassing the efficacy of CD244-deficient BMDM alone or a combination of WT BMDM and anti-PD-L1 antibody. Together, our data provide a mechanistic and clinical basis for considering CD244-deficient macrophages as a novel therapeutic modality, either as a standalone treatment or in conjunction with checkpoint blockade therapies.

CD244 (SLAMF4; 2B4) belongs to the SLAM family receptors (SFR), a group of nine transmembrane receptors that play crucial roles in immune regulation [41–43]. All SFRs, except CD244 and CD48, are homotypic receptors that engage in either *trans*-interactions on neighboring cells or *cis*-interactions on the same hematopoietic cells. CD244 interacts with CD48, the expression of which is restricted to hematopoietic cells. The role of SFRs on monocyte-lineage cells has only recently been recognized [21, 44–46]. For example, Chen et al. demonstrated that SLAMF7 promotes phagocytosis of hematopoietic tumor cells by macrophages in the absence of SIRPα-CD47 interactions [47]. Additionally, Li et al. found that the gene deletion of CD48, the ligand of CD244, promotes LRP1-dependent, CD47-independent phagocytic pathways in macrophages against hematopoietic cells, but not solid tumors [46]. To expand upon these findings, our melanoma data using CD244 KO and CD244^fl/fl^LysM^cre^ conditional knockout mice provide direct evidence that CD244 also inhibits anti-tumor immunity in solid tumors by restricting macrophage phagocytosis through inhibiting maturation of monocytes to Ly6C^low^ macrophages.

Mechanistically, we present for the first time that enhanced autophagy in CD244-deficient monocyte-lineage cells promotes the differentiation of anti-tumorigenic Ly6C^low^ macrophages within the tumor microenvironment. Our observations were further supported by increased autophagy signatures in CD244-negative monocyte-lineage cells from human melanoma patients. Autophagy has been recognized as crucial for macrophage differentiation and the acquisition of phagocytic functions, as evidenced by experiments utilizing siRNAs targeting Beclin-1 or other autophagy inhibitors, which resulted in a significant reduction in macrophage differentiation [36, 37]. Likewise, increased interaction between Beclin-1, Vps-34, and CD244 under CD244-CD48 ligation has been previously observed in BMDMs, but the implications of this interaction on cellular homeostasis or immunological responses remained unclear until now [35]. Based on our data and prior studies [35], we hypothesize that the inhibition of autophagy and macrophage differentiation seen by CD244 is likely resulted from hijacking Beclin-1 and Vps-34.

Although the role of autophagy in tumorigenesis remains controversial, it is recognized that autophagy plays a pivotal role in promoting the anti-tumorigenic functions of macrophages. Inhibition of the mTOR pathway with rapamycin has been shown to stimulate a pro-inflammatory macrophage phenotype, characterized by a substantial increase in IL-6 expression and a decrease in IL-10 expression [48]. Furthermore, autophagy is essential for efficient antigen cross-presentation for activating CD8 T cells. Previous studies have demonstrated that CD103^+^ dendritic cells (DCs) within tumors can activate CD8 T cells through cross-presentation [49, 50], and emerging evidence suggests that M1 macrophages can function in a similar manner [51]. Deficiencies in autophagy-related genes such as Beclin-1, Atg5, Atg7, or GCN2 in bone marrow-derived DCs have been shown to impair the cross-presentation of antigens derived from cells infected with a yellow fever virus vaccine strain [52]. Additionally, primary CD8α^+^ DCs, specialized in cross-presenting antigens, exhibit heightened autophagy compared to CD8α^−^ DCs, highlighting the active role of autophagy in specialized cross-presenting DCs [53]. Altogether, these data supported the importance of the autophagy pathways regulated by CD244 in macrophages for the cross-presentation and differentiation of macrophages.

To investigate the underlying cause of the significant increase in CD244 expression on monocytes/macrophages following tumor induction, we conducted a comprehensive analysis utilizing public mouse single-cell RNA sequencing (scRNA-seq) data and performed ex vivo experiments. Our findings revealed that endoplasmic reticulum (ER) stress induces the upregulation of CD244 in monocytes. Within the tumor microenvironment, characterized by factors such as hypoxia, nutrient deprivation, low pH, and elevated cytokines and growth factors, infiltrating monocytes and neutrophils experience ER stress. In response to this stress, myeloid cells activate the unfolded protein response (UPR) to enhance cell survival and adaptation. Consequently, ER stress plays a vital protective role in the survival of myeloid-derived suppressor cells (MDSCs), thereby exacerbating the immunosuppressive environment within tumors [54]. Our findings also provide insights into the association between CD244 expression and signatures of immunosuppressive monocytes, as previously reported in mouse models of head and neck squamous cell carcinoma [21]. This suggests that ER stress-mediated CD244 expression restrains monocytes in an immunosuppressive state. Notably, decreased CD244 expression has been observed in monocytes isolated from patients with systemic lupus erythematosus (SLE), an autoimmune disease in which monocyte differentiation and polarization into pro-inflammatory macrophages have been implicated in disease pathogenesis [45, 55]. In contrast, monocytic-myeloid-derived suppressor cells (M-MDSCs) have exhibited a protective role in autoimmune diseases [56]. Based on our study, we hypothesize that CD244 might restrain monocyte differentiation in autoimmune diseases, thereby curtailing disease progression. Our findings provide new insights into the potential involvement of CD244 in regulating the balance between immunosuppressive monocytes and pro-inflammatory macrophages in the context of both autoimmune diseases and cancers. Further investigations are warranted to fully unravel the underlying mechanisms involved.

Intrigued by our compelling results in the combination therapy involving CD244-deficient macrophages and anti-PD-L1 antibody, which demonstrated a significant reduction in tumor size compared to the control groups, we assert that the impact of CD244 removal in macrophages profoundly transforms the tumor immune microenvironment, holding clinical significance. Beyond its combination with immune checkpoint inhibitors, this approach opens avenues for the utilization of various therapeutic strategies targeting solid tumors. In melanoma, for instance, where mutations in BRAF are prevalent in 40–50% of cases, extensive studies to inhibit mutated BRAF using drugs like vemurafenib and dabrafenib have been conducted in clinical setting. Previous studies indicate that simultaneous inhibition of BRAF and MEK or MAPK induces pyroptosis in tumor cells, thereby enhancing the immune responses [57]. Furthermore, the response rate to these inhibitors is intricately linked to the patient’s tumor microenvironment [58]. Therefore, experiments are underway to investigate whether the combined administration of CD244-deficient macrophages with targeted therapies, whose efficacy are influenced by the tumor immune microenvironment, such as BRAF inhibitors, can amplify anti-tumor immune responses through the enhancement of tumor antigen-specific CD8 T cell responses.

Several limitations were noted in this study. Firstly, while LysM-cre primarily targets monocytes/macrophages, it also efficiently affects neutrophils and has a lesser impact on DCs [59, 60]. Although we did not observe significant alterations in CD244 expression levels or cell proportions within these cell populations in CD244^fl/fl^LysM^cre^ mice, the observed effects might be contributed to by other cell types in vivo. However, at least in our experiments, we did not find a significant contribution of neutrophils and DCs to phagocytosis and antigen-specific T cell activation. Nevertheless, we cannot exclude the possibility that CD244 may modulate other myeloid cell types in a *cis*- or *trans*-manner to regulate the tumor microenvironment. Secondly, considering the role of M1 macrophages in the development and progression of various tumor types, it is essential to determine if the anti-tumorigenic function of CD244-negative monocyte-lineage cells holds true for other tumor types as well. Although scRNA-seq data have confirmed the involvement of CD244 in the differentiation and function of monocytes/macrophages in patients with other solid tumors (colorectal cancer, non-small cell lung cancer, and glioblastoma), further studies are necessary to confirm the association with tumor progression. These investigations could provide valuable insights into the therapeutic potential of targeting CD244 on monocyte-lineage cells for treating a wide range of human cancers.

In conclusion, our study highlights the novel role of CD244 on monocytes/macrophages, which restrains the maturation of anti-tumorigenic macrophages and dampens the antigen-specific activation of T cells in melanoma. Our findings propose that CD244-deficient macrophages could potentially serve as therapeutic agent in immunologically “cold” tumors, reinvigorating exhausted T cells into memory cells when combined with checkpoint inhibitors. Furthermore, CD244 expression in monocyte-lineage cells acts as a predictive marker in cancer patients. Our study sheds light on an unexplored aspect of immune regulation in cancer and has important implications for the development of novel immunotherapeutic strategies, in conjunction with checkpoint blockade to effectively enhance tumor-antigen specific T cell immunity.

### Electronic supplementary material

Below is the link to the electronic supplementary material.

Supplementary Material 1

Supplementary Material 2

Supplementary Material 3

Supplementary Material 4

Supplementary Material 5

## Data Availability

All data pertaining to this study have been either presented in the paper or included in the Supplementary Materials. Any resources generated during this research can be obtained upon request from the lead author, subject to the completion of a material transfer agreement. The accession number for the data presented here includes an scRNA-seq dataset with accession ID: GSE121861 (encompassing mouse syngeneic tumor, SCP398; human melanoma, SCP1162; human CRC, GSE127465; human NSCLC, GSE131928; human GBM). Additionally, RNA-seq datasets from melanoma patients have been retrieved from The Cancer Genome Atlas Program (TCGA) database (TCGA-SKCM; https://portal.gdc.cancer.gov) and the GEO database with accession ID: GSE91061. All source codes have been archived at 10.5281/zenodo.6844694 and are publicly accessible from the date of publication.

## Abbreviations

ARG-1: Arginase-1
ATCC: American Type Culture Collection
BM: Bone marrow
BMDM: Bone marrow-derived macrophages
cDNA: Complementary DNA
CFSE: Carboxyfluorescein succinimidyl ester
CD244^fl/fl^: CD244 Floxed mice (control), CD244^fl/fl^-LysM-cre^−/^
CD244^fl/fl^LysM^cre^: CD244 Conditional KO mice; CD244^fl/fl^-LysM-cre^+/^
CMPs: Common myeloid progenitors
CRC: Colorectal cancer
CTLA-4: Cytotoxic T-lymphocyte-associated antigen-4
DC: Dendritic cell
DEGs: Differentially expressed genes
ER: Endoplasmic reticulum
FPKM: Fragments per kilobase of transcript per million mapped reads
GBM: Glioblastoma
GEO: Gene Expression Omnibus
GM-CSF: Granulocyte-macrophage colony stimulating factor
GMPs: Granulocyte/macrophage progenitors
GOBPs: Gene ontology biological processes
GSEA: Gene set enrichment analysis
HVGs: Highly variable genes
IFN: Interferon
IL: Interleukin
iNOS: Inducible nitric oxide synthase
LAG-3: Lymphocyte-activation gene-3
M-CSF: Macrophage colony-stimulating factor
mAb: Monoclonal antibody
MDSCs: Myeloid-derived suppressor cells
NK cell: Natural killer cell
NSCLC: Non-small cell lung cancer
OVA: Ovalbumin
PCs: Principal components
PD-1: Programmed cell death protein 1
PD-L1: Programmed cell death ligand-1
RT: Room temperature
scRNA-seq: Single cell RNA sequencing
SFRs: SLAM family receptors
SLE: Systemic lupus erythematosus
TAM: Tumor associated macrophage
TCGA: The Cancer Genome Atlas
TDLN: Tumor draining lymph node
TGF-b: Transforming growth factor-b
THG: Thapsigargin
TIGIT: T cell immunoreceptor with Ig and ITIM domains
TILs: Tumor infiltrating lymphocytes
TIM-3: T-cell immunoglobulin and mucin-domain containing-3
TME: Tumor microenvironment
TUDCA: Tauroursodeoxycholic acid
UMAP: Uniform manifold approximation and projection
UMI: Unique molecular identifier
wKO: Whole knockout
WT: Wild-type

## References

[1] Lee KM, Chuang E, Griffin M, Khattri R, Hong DK, Zhang WG (1998). Molecular basis of T cell inactivation by CTLA-4. Science.

[2] Cai GF, Freeman GJ (2009). The CD160, BTLA, LIGHT/HVEM pathway: a bidirectional switch regulating T-cell activation. Immunol Rev.

[3] Boussiotis VA (2016). Molecular and biochemical aspects of the PD-1 checkpoint pathway. N Engl J Med.

[4] Anderson AC, Joller N, Kuchroo VK, Lag-3. Tim-3, and TIGIT: co-inhibitory receptors with Specialized functions in Immune Regulation. Immunity. 2016;44(5):989–1004. 10.1016/j.immuni.2016.05.001 PMC4942846 27192565

[5] Calabro L, Danielli R, Sigalotti L, Maio M (2010). Clinical studies with Anti-CTLA-4 antibodies in non-melanoma indications. Semin Oncol.

[6] Brahmer JR, Tykodi SS, Chow LQM, Hwu WJ, Topalian SL, Hwu P (2012). Safety and activity of Anti-PD-L1 antibody in patients with Advanced Cancer. N Engl J Med.

[7] Topalian SL, Hodi FS, Brahmer JR, Gettinger SN, Smith DC, McDermott DF (2012). Safety, Activity, and Immune correlates of Anti-PD-1 antibody in Cancer. N Engl J Med.

[8] Tawbi HA, Schadendorf D, Lipson EJ, Ascierto PA, Matamala L, Castillo Gutierrez E (2022). Relatlimab and Nivolumab versus Nivolumab in Untreated Advanced Melanoma. N Engl J Med.

[9] Wherry EJ, Kurachi M (2015). Molecular and cellular insights into T cell exhaustion. Nat Rev Immunol.

[10] Sade-Feldman M, Yizhak K, Bjorgaard SL, Ray JP, de Boer CG, Jenkins RW (2018). Defining T Cell States Associated with response to Checkpoint Immunotherapy in Melanoma. Cell.

[11] Shah P, Cuoco M, Su MJ, Melms J, Leeson R, Kanodia A (2020). A cancer cell program promotes T-cell exclusion and resistance to checkpoint blockade. Cancer Res.

[12] Kalbasi A, Ribas A (2020). Tumour-intrinsic resistance to immune checkpoint blockade. Nat Rev Immunol.

[13] Gettinger S, Choi JM, Hastings K, Truini A, Datar I, Sowell R (2017). Impaired HLA Class I Antigen Processing and Presentation as a mechanism of Acquired Resistance to Immune checkpoint inhibitors in Lung Cancer. Cancer Discov.

[14] Veglia F, Perego M, Gabrilovich D (2018). Myeloid-derived suppressor cells coming of age. Nat Immunol.

[15] Weber R, Fleming V, Hu X, Nagibin V, Groth C, Altevogt P (2018). Myeloid-derived suppressor cells hinder the anti-cancer activity of Immune Checkpoint inhibitors. Front Immunol.

[16] Garni-Wagner BA, Purohit A, Mathew PA, Bennett M, Kumar V (1993). A novel function-associated molecule related to non-MHC-restricted cytotoxicity mediated by activated natural killer cells and T cells. J Immunol.

[17] Lee KM, McNerney ME, Stepp SE, Mathew PA, Schatzle JD, Bennett M (2004). 2B4 acts as a non-major histocompatibility complex binding inhibitory receptor on mouse natural killer cells. J Exp Med.

[18] Wu Y, Kuang DM, Pan WD, Wan YL, Lao XM, Wang D (2013). Monocyte/macrophage-elicited natural killer cell dysfunction in hepatocellular carcinoma is mediated by CD48/2B4 interactions. Hepatology.

[19] Mittal R, Chen CW, Lyons JD, Margoles LM, Liang Z, Coopersmith CM (2015). Murine lung cancer induces generalized T-cell exhaustion. J Surg Res.

[20] Goding SR, Wilson KA, Xie Y, Harris KM, Baxi A, Akpinarli A (2013). Restoring Immune function of Tumor-Specific CD4(+) T cells during recurrence of Melanoma. J Immunol.

[21] Agresta L, Lehn M, Lampe K, Cantrell R, Hennies C, Szabo S et al. CD244 represents a new therapeutic target in head and neck squamous cell carcinoma. J Immunother Cancer. 2020;8(1). 10.1136/jitc-2019-000245 PMC7174077 32217758

[22] Kumar MP, Du J, Lagoudas G, Jiao Y, Sawyer A, Drummond DC (2018). Analysis of single-cell RNA-Seq identifies cell-cell communication Associated with Tumor characteristics. Cell Rep.

[23] Pelka K, Hofree M, Chen JH, Sarkizova S, Pirl JD, Jorgji V (2021). Spatially organized multicellular immune hubs in human colorectal cancer. Cell.

[24] Zilionis R, Engblom C, Pfirschke C, Savova V, Zemmour D, Saatcioglu HD (2019). Single-cell transcriptomics of human and mouse lung cancers reveals conserved myeloid populations across individuals and species. Immunity.

[25] Neftel C, Laffy J, Filbin MG, Hara T, Shore ME, Rahme GJ (2019). An Integrative Model of Cellular States, plasticity, and Genetics for Glioblastoma. Cell.

[26] Stuart T, Butler A, Hoffman P, Hafemeister C, Papalexi E, Mauck WM (2019). Comprehensive Integration of Single-Cell Data. Cell.

[27] Herwig R, Hardt C, Lienhard M, Kamburov A (2016). Analyzing and interpreting genome data at the network level with ConsensusPathDB. Nat Protoc.

[28] Network CGA (2015). Genomic classification of cutaneous melanoma. Cell.

[29] Riaz N, Havel JJ, Makarov V, Desrichard A, Urba WJ, Sims JS (2017). Tumor and Microenvironment Evolution during Immunotherapy with Nivolumab. Cell.

[30] Newman AM, Liu CL, Green MR, Gentles AJ, Feng WG, Xu Y (2015). Robust enumeration of cell subsets from tissue expression profiles. Nat Methods.

[31] Budd RC, Cerottini JC, Horvath C, Bron C, Pedrazzini T, Howe RC (1987). Distinction of virgin and memory Lymphocytes-T stable Acquisition of the Pgp-1 glycoprotein concomitant with antigenic-stimulation. J Immunol.

[32] Li YH, Zhang Y, Pan G, Xiang LX, Luo DC, Shao JZ. Occurrences and functions of Ly6C(hi) and Ly6C(lo) macrophages in Health and Disease. Front Immunol. 2022;13. 10.3389/fimmu.2022.901672 PMC9189283 35707538

[33] Liu Y, Cao X (2015). The origin and function of tumor-associated macrophages. Cell Mol Immunol.

[34] Bronte V, Brandau S, Chen SH, Colombo MP, Frey AB, Greten TF (2016). Recommendations for myeloid-derived suppressor cell nomenclature and characterization standards. Nat Commun.

[35] Chaudhary A, Leite M, Kulasekara BR, Altura MA, Ogahara C, Weiss E (2016). Human diversity in a cell surface receptor that inhibits autophagy. Curr Biol.

[36] Jacquel A, Obba S, Boyer L, Dufies M, Robert G, Gounon P (2012). Autophagy is required for CSF-1-induced macrophagic differentiation and acquisition of phagocytic functions. Blood.

[37] Zhang Y, Morgan MJ, Chen K, Choksi S, Liu ZG (2012). Induction of autophagy is essential for monocyte-macrophage differentiation. Blood.

[38] Obba S, Hizir Z, Boyer L, Selimoglu-Buet D, Pfeifer A, Michel G (2015). The PRKAA1/AMPK1 pathway triggers autophagy during CSF1-induced human monocyte differentiation and is a potential target in CMML. Autophagy.

[39] Kleffel S, Posch C, Barthel SR, Mueller H, Schlapbach C, Guenova E (2015). Melanoma Cell-intrinsic PD-1 receptor functions promote Tumor Growth. Cell.

[40] Lin H, Wei S, Hurt EM, Green MD, Zhao L, Vatan L (2018). Host expression of PD-L1 determines efficacy of PD-L1 pathway blockade-mediated tumor regression. J Clin Invest.

[41] Veillette A (2006). Immune regulation by SLAM family receptors and SAP-related adaptors. Nat Rev Immunol.

[42] Cannons JL, Tangye SG, Schwartzberg PL (2011). SLAM family receptors and SAP adaptors in immunity. Annu Rev Immunol.

[43] Calpe S, Wang NH, Romero X, Berger SB, Lanyi A, Engel P (2008). The SLAM and SAP gene families control innate and adaptive immune responses. Adv Immunol.

[44] Straub C, Neulen ML, Viertlboeck BC, Gobel TW (2014). Chicken SLAMF4 (CD244, 2B4), a receptor expressed on thrombocytes, monocytes, NK cells, and subsets of alphabeta-, gammadelta- T cells and B cells binds to SLAMF2. Dev Comp Immunol.

[45] Mak A, Thornhill SI, Lee HY, Lee B, Poidinger M, Connolly JE (2018). Brief report: decreased expression of CD244 (SLAMF4) on monocytes and platelets in patients with systemic lupus erythematosus. Clin Rheumatol.

[46] Li D, Xiong W, Wang YD, Feng J, He YX, Du J et al. SLAMF3 and SLAMF4 are immune checkpoints that constrain macrophage phagocytosis of hematopoietic tumors. Sci Immunol. 2022;7(67). 10.1126/sciimmunol.abj5501 35061505

[47] Chen J, Zhong MC, Guo HJ, Davidson D, Mishel S, Lu Y (2017). SLAMF7 is critical for phagocytosis of haematopoietic tumour cells via Mac-1 integrin. Nature.

[48] Chen W, Ma T, Shen XN, Xia XF, Xu GD, Bai XL (2012). Macrophage-Induced Tumor Angiogenesis is regulated by the TSC2-mTOR pathway. Liver Transpl.

[49] Sharma MD, Rodriguez PC, Koehn BH, Baban B, Cui Y, Guo G (2018). Activation of p53 in immature myeloid precursor cells controls differentiation into Ly6c(+)CD103(+) Monocytic Antigen-presenting cells in tumors. Immunity.

[50] Broz M, Binnewies M, Boldajipour B, Nelson A, Pollock J, Erle D et al. Dissecting the tumor myeloid compartment reveals rare activating antigen presenting cells, critical for T cell immunity. Cancer Immunol Res. 2015;3(10). 10.1016/j.ccell.2014.09.007 PMC4254577 25446897

[51] Muntjewerff EM, Meesters LD, van den Bogaart G. Antigen Cross-presentation by macrophages. Front Immunol. 2020;11. 10.3389/fimmu.2020.01276 PMC7360722 32733446

[52] Ravindran R, Khan N, Nakaya HI, Li SZ, Loebbermann J, Maddur MS (2014). Vaccine activation of the Nutrient Sensor GCN2 in dendritic cells enhances Antigen Presentation. Science.

[53] Mintern JD, Macri C, Chin WJ, Panozza SE, Segura E, Patterson NL (2015). Differential use of autophagy by primary dendritic cells specialized in cross-presentation. Autophagy.

[54] Lou XL, Gao DY, Yang LY, Wang Y, Hou YQ. Endoplasmic reticulum stress mediates the myeloid-derived immune suppression associated with cancer and infectious disease. J Transl Med. 2023;21(1). 10.1186/s12967-022-03835-4 PMC9809056 36593497

[55] Korman BD, Huang CC, Skamra C, Wu P, Koessler R, Yao D (2014). Inflammatory expression profiles in monocyte-to-macrophage differentiation in patients with systemic lupus erythematosus and relationship with atherosclerosis. Arthritis Res Ther.

[56] Ji J, Li P, Shen C, Dou H, Wang T, Shi L (2019). MDSCs: friend or foe in systemic lupus erythematosus. Cell Mol Immunol.

[57] Erkes DA, Cai W, Sanchez IM, Purwin TJ, Rogers C, Field CO (2020). Mutant BRAF and MEK inhibitors regulate the Tumor Immune Microenvironment via Pyroptosis. Cancer Discov.

[58] Lelliott EJ, McArthur GA, Oliaro J, Sheppard KE (2021). Immunomodulatory effects of BRAF, MEK, and CDK4/6 inhibitors: implications for combining targeted therapy and Immune Checkpoint Blockade for the Treatment of Melanoma. Front Immunol.

[59] Abram CL, Roberge GL, Hu Y, Lowell CA (2014). Comparative analysis of the efficiency and specificity of myeloid-cre deleting strains using ROSA-EYFP reporter mice. J Immunol Methods.

[60] McCubbrey AL, Allison KC, Lee-Sherick AB, Jakubzick CV, Janssen WJ. Promoter specificity and efficacy in conditional and Inducible Transgenic targeting of lung macrophages. Front Immunol. 2017;8. 10.3389/fimmu.2017.01618 PMC5705560 29225599

