# Supplementary figures and images for: Targeted deletion of CD244 on monocytes promotes differentiation into anti-tumorigenic macrophages and potentiates PD-L1 blockade in melanoma
Source: Mol Cancer. 2024 Feb 29;23:45. doi: 10.1186/s12943-024-01936-w (PMC10903025; doi:10.1186/s12943-024-01936-w)

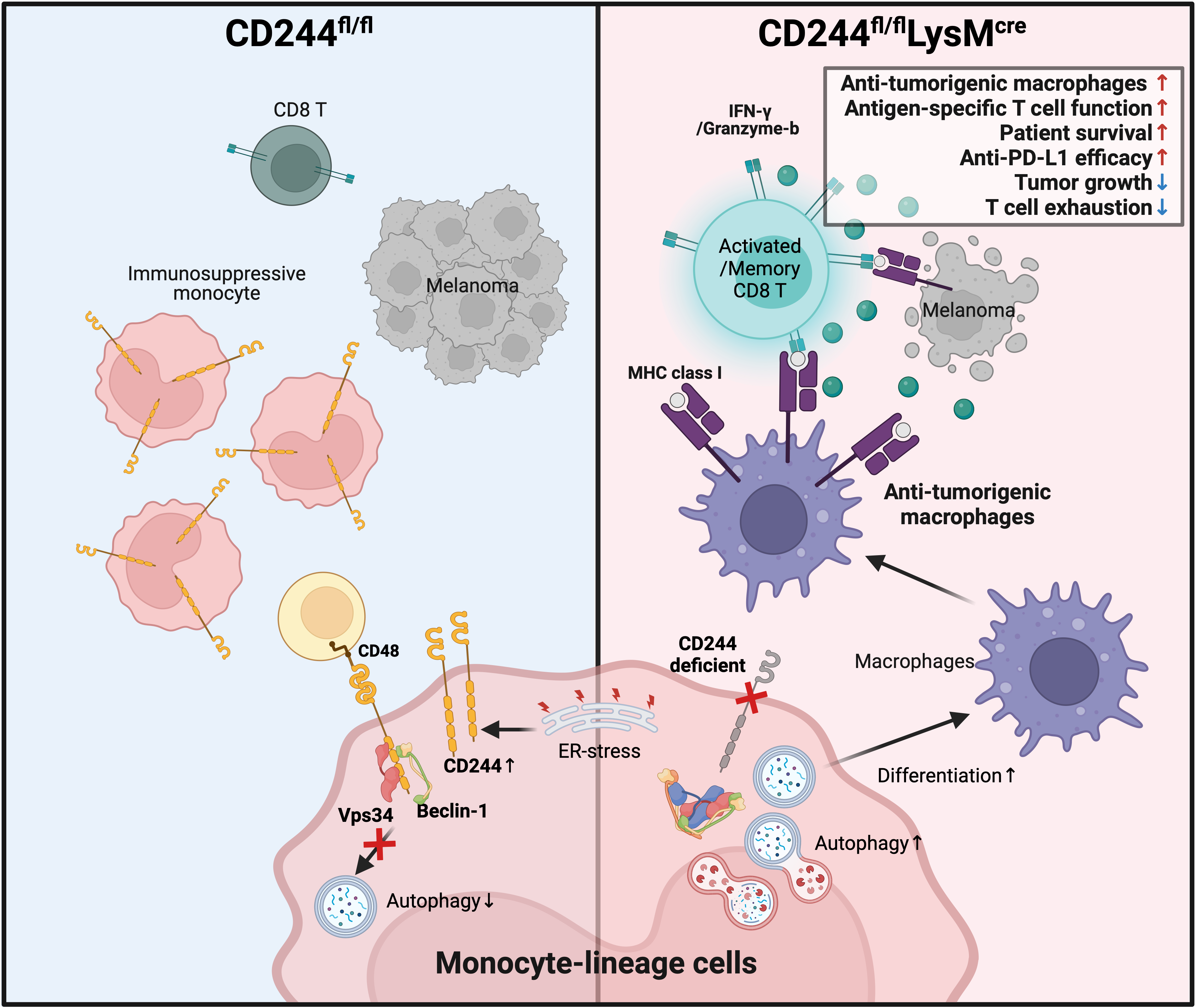

Supplement: Supplementary file 1 — Supplementary Material 1 [file 12943_2024_1936_MOESM1_ESM.jpg]
