# Supplementary material for: Targeted deletion of CD244 on monocytes promotes differentiation into anti-tumorigenic macrophages and potentiates PD-L1 blockade in melanoma
Source: Mol Cancer. 2024 Feb 29;23:45. doi: 10.1186/s12943-024-01936-w (PMC10903025; doi:10.1186/s12943-024-01936-w)
Supplement: Supplementary file 5 — Supplementary Material 5 [file 12943_2024_1936_MOESM5_ESM.pdf]

**Fig. S1. Immune cell population in CD244$^{-/-}$ mice, and the generation of monocyte-lineage specific CD244 conditional KO mice.**

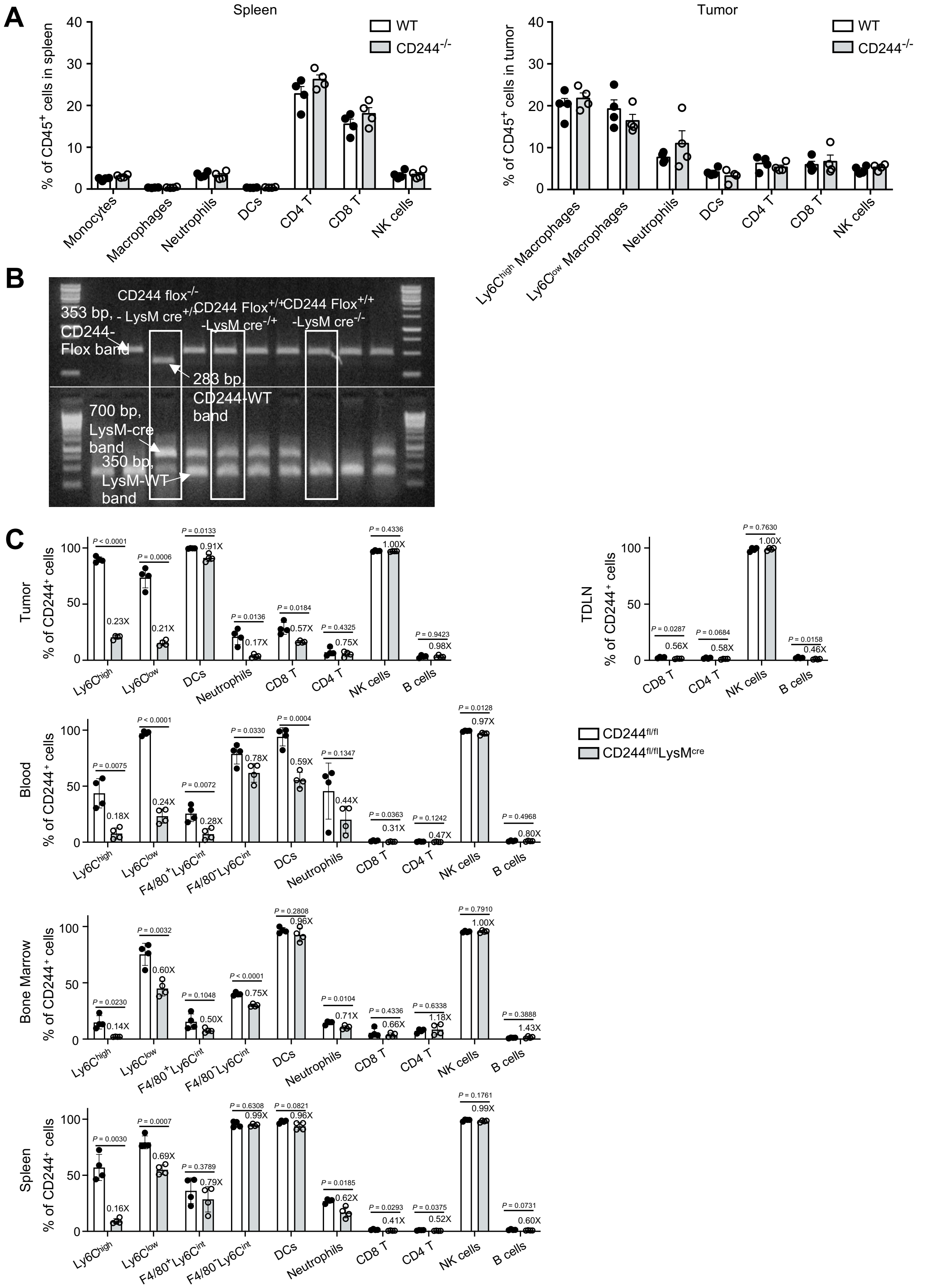

**(A)** 1 x $10^6$ of B16F10 cells were subcutaneously injected into the right flank of both WT and $CD244^{-/-}$ mice. The proportion of immune cell subtypes in the spleen **(left)** and tumor **(right)** was evaluated 14 days after tumor inoculation. **(B)** The genotyping results for $CD244^{fl/fl}$ and $CD244^{fl/fl}$LysM-$cre^{-/+}$ mice are presented. **(C)** CD244 deletion in $CD244^{fl/fl}LysM^{cre}$ mice was verified through flow cytometry analysis in comparison to $CD244^{fl/fl}$ mice. CD244 expression in myeloid and lymphoid cells across various tissues, including the tumor, blood, bone marrow, spleen and tumor-draining lymph node (TDLN). Numbers above $CD244^{fl/fl}LysM^{cre}$ bars indicated the fold change of CD244-expressing cell proportions in $CD244^{fl/fl}LysM^{cre}$ compared to $CD244^{fl/fl}$. Significance was indicated as *P*-value, and the statistical analysis was performed using unpaired Student's *t*-test **(C)**.

**Fig. S2. The proportions of tumor-infiltrating lymphocyte populations and the expression of IFN-γ in CD8/CD4 T cells in the tumor-draining lymph nodes (TDLN) from CD244$^{fl/fl}$ and CD244$^{fl/fl}$LysM$^{cre}$ mice.**

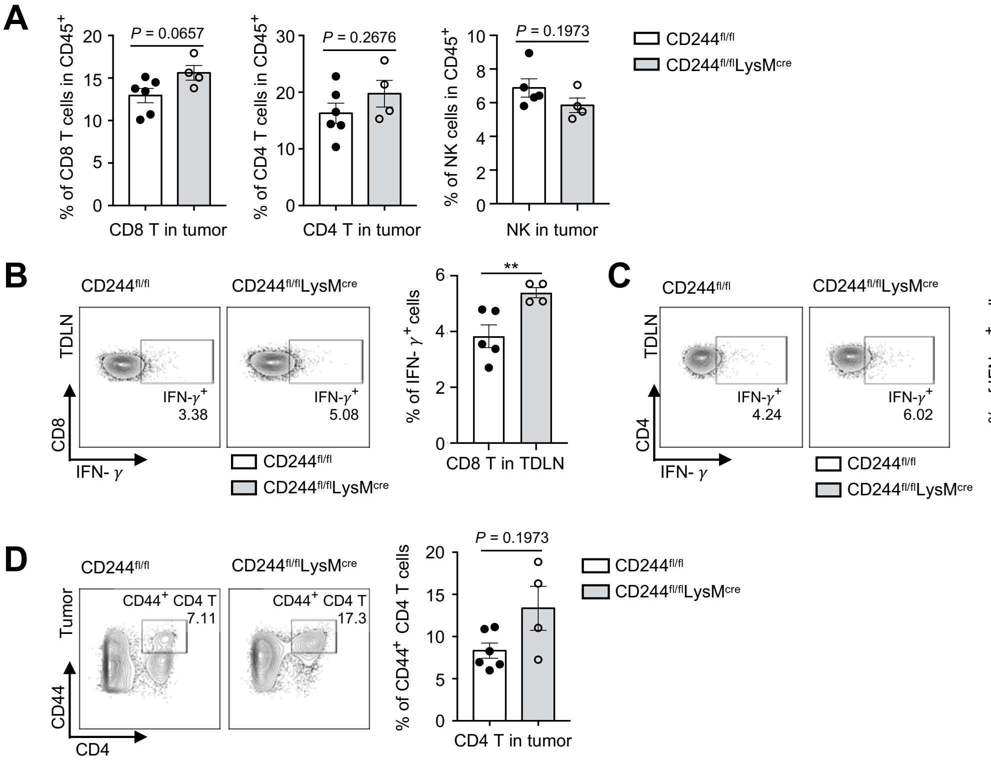


**(A-D)** The CD45$^+$ cell population in the tumor and the tumor-draining lymph nodes (TDLN) were analyzed using flow cytometry 14 days after inoculating 1 x $10^6$ B16F10 cells into CD244$^{fl/fl}$ and CD244$^{fl/fl}$LysM$^{cre}$ mice. The proportion of CD8 T, CD4 T, and NK cells in the total CD45$^+$ cell subsets were assessed within the tumor **(A)**, The expression of IFN-γ in CD8 T cells **(B)** and CD4 T cells **(C)** in the TDLN was determined. Additionally, the proportion of CD44$^+$ cells among CD4 T cells in the tumor was examined **(D)**. Significance was indicated as $*P < 0.05$; $**P < 0.01$, and the statistical analysis was performed using an unpaired Student's *t*-test. The data provided is representative of four **(A)** and two **(B-D)** independent experiments for panels.

**Fig. S3. Analysis of myeloid cell population, caspase-3 expression, MHC class II expression in monocytes/macrophages within the tumor and in the bone marrow-derived macrophages (BMDM) of CD244$^{fl/fl}$ and CD244$^{fl/fl}$LysM$^{cre}$ mice.**

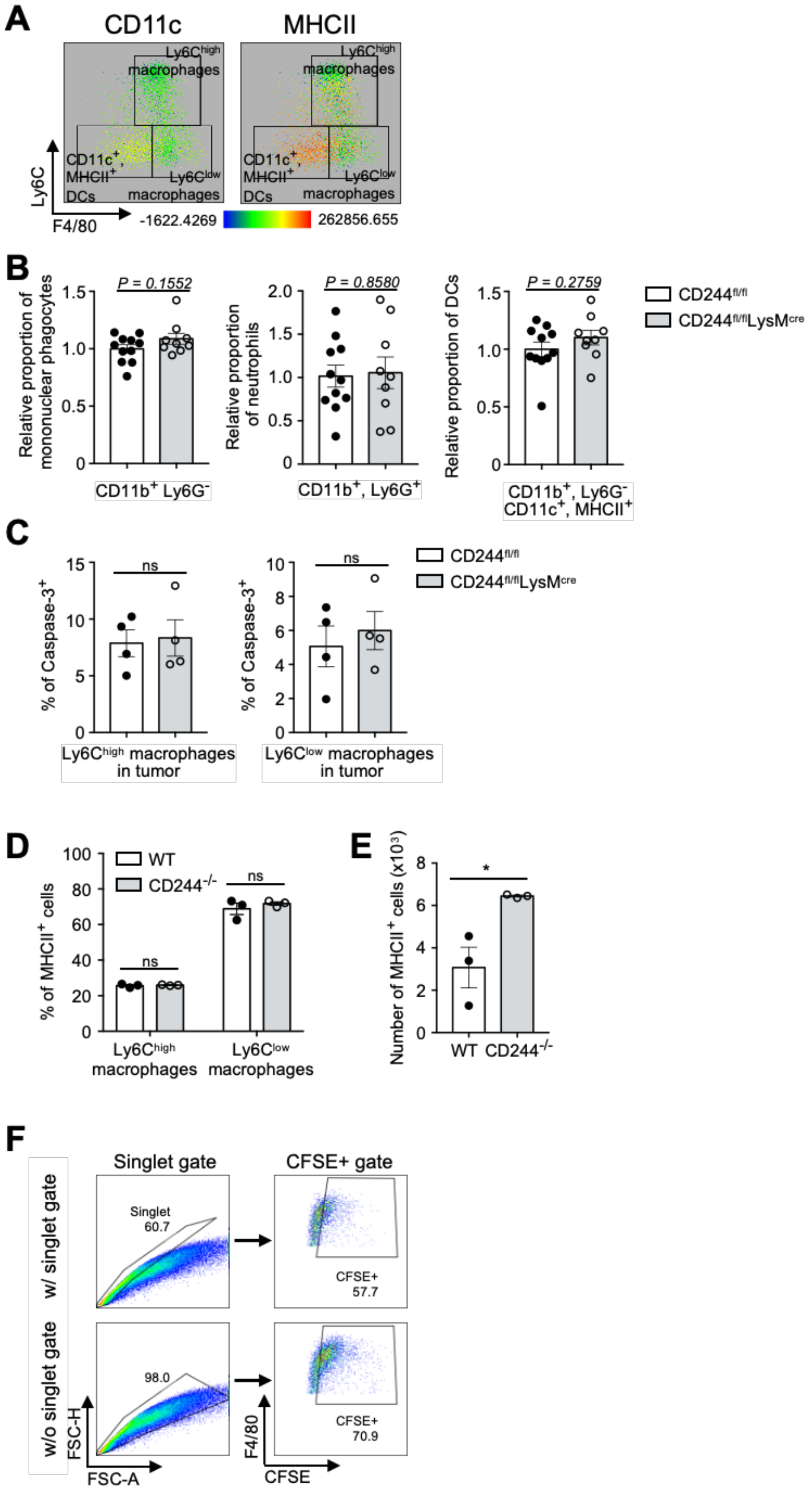

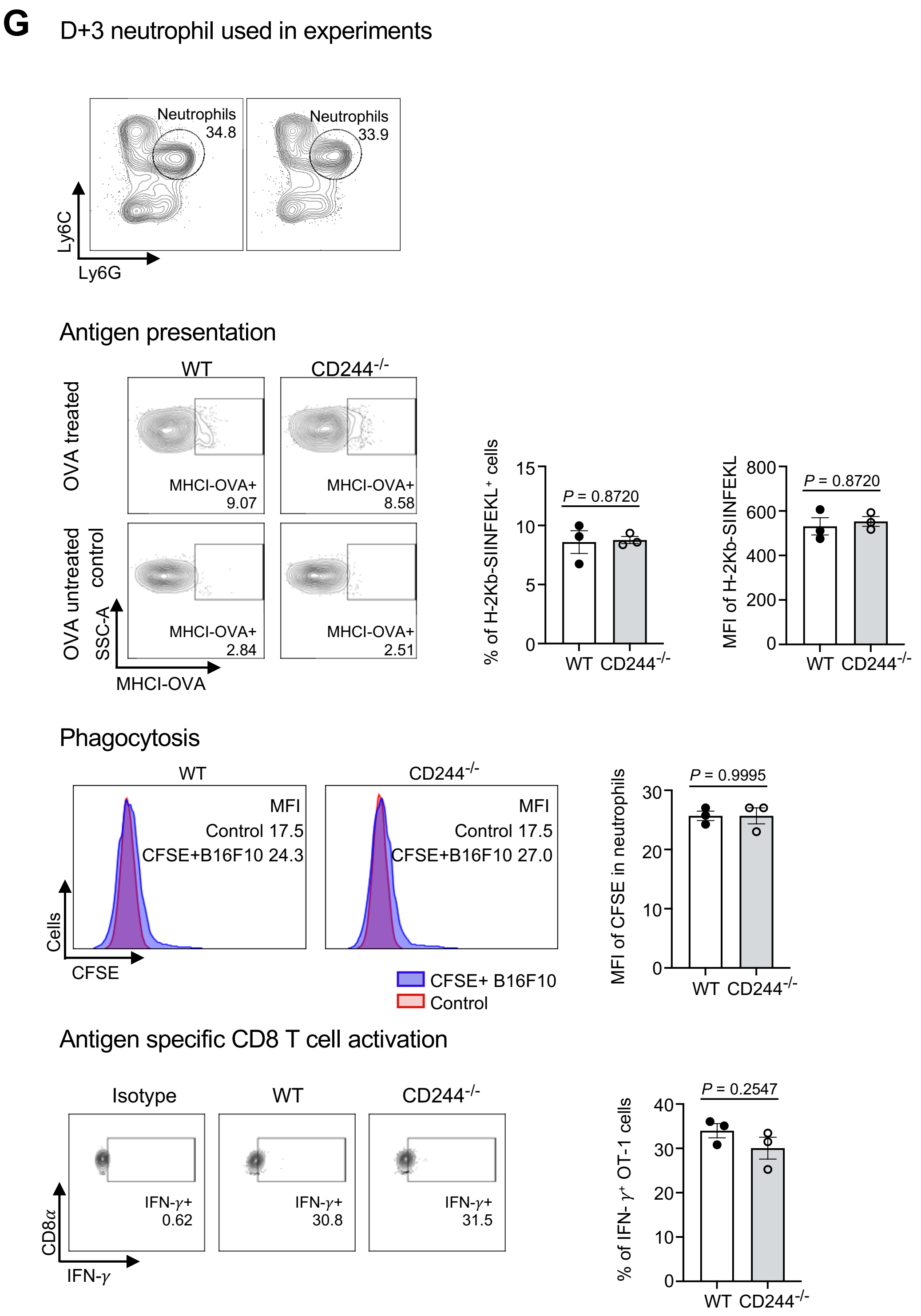
G
D+3 neutrophil used in experiments
Neutrophils
34.8
Neutrophils
33.9
Ly6C
Ly6G
Antigen presentation
WT
CD244-/-
OVA treated
OVA untreated control
MHCI-OVA+
9.07
MHCI-OVA+
8.58
MHCI-OVA+
2.84
MHCI-OVA+
2.51
SSC-A
MHCI-OVA
% of H-2Kb-SIINFEKL+ cells
P = 0.8720
WT CD244-/-
MFI of H-2Kb-SIINFEKL
P = 0.8720
WT CD244-/-
Phagocytosis
WT
CD244-/-
MFI
Control 17.5
CFSE+B16F10 24.3
MFI
Control 17.5
CFSE+B16F10 27.0
Cells
CFSE
CFSE+ B16F10
Control
MFI of CFSE in neutrophils
P = 0.9995
WT CD244-/-
Antigen specific CD8 T cell activation
Isotype
WT
CD244-/-
IFN-γ+
0.62
IFN-γ+
30.8
IFN-γ+
31.5
CD8α
IFN-γ
% of IFN-γ+ OT-1 cells
P = 0.2547
WT CD244-/-

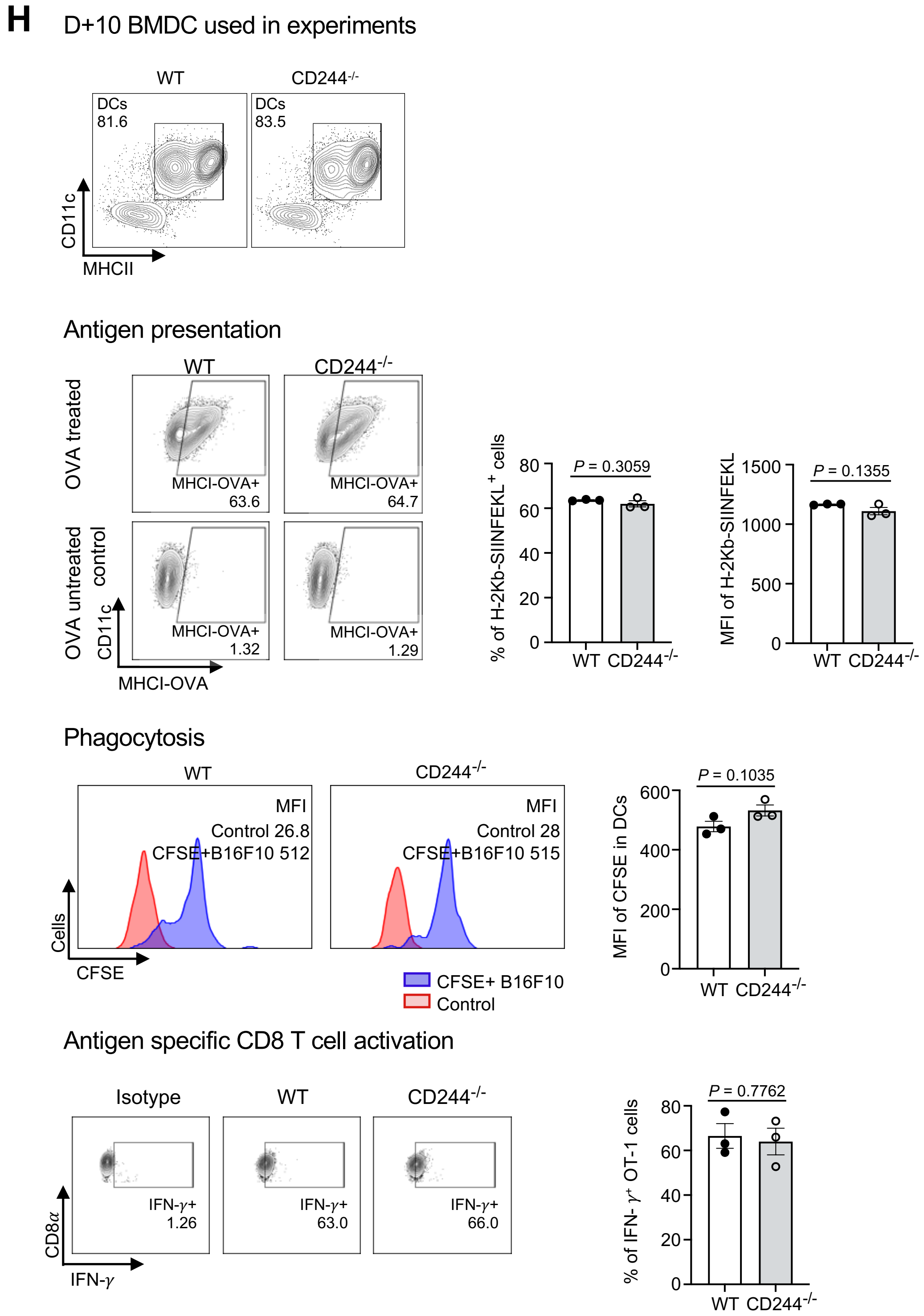


**(A-C)** After injecting B16F10 cells into CD244$^{fl/fl}$ and CD244$^{fl/fl}$LysM$^{cre}$ mice, the proportions of myeloid subpopulations and caspase-3 expression were assessed within tumor mass 14 days later. **(A)** Flow cytometry plots showing the high expression of CD11c and MHCII in Ly6C$^{-}$, F4/80$^{-}$ population, indicating the presence of DCs. **(B)** Relative proportion of myeloid cells (CD11b$^{+}$) within CD45$^{+}$ subsets, Ly6G$^{+}$ neutrophils and DCs (CD11c$^{+}$MHC-II$^{+}$) within

$CD11b^+$ cells were determined. **(C)** Caspase-3 expression in $Ly6C^{high}$ macrophages and $Ly6C^{low}$ macrophages was evaluated. **(D-E)** Bone marrow cells were cultured with M-CSF and whole OVA protein, and MHC-II expression was measured on day 3. The proportion **(D)** and absolute number **(E)** of MHC-II expressing cells in WT and $CD244^{-/-}$ BMDMs were analyzed. **(F)** Flow cytometry plots illustrating the gating strategy to exclude non-specific binding of BMDMs and $CFSE^+$ B16F10 cells. **(G-H)** Antigen presentation, phagocytosis, antigen specific CD8 T cell activation property of neutrophils **(G)** and DCs **(H)** were examined. To obtain neutrophils, we differentiated bone marrow (BM) cells from WT or $CD244^{-/-}$ mice with GM-CSF for 3 days. For preparing DCs, we differentiated bone marrow (BM) cells from WT or $CD244^{-/-}$ mice with GM-CSF and cultured them for 10 days. To examine the role of CD244 in the ability of MHC class I-mediated antigen presentation in neutrophils and DCs, we co-cultured $Ly6C^+Ly6G^+$ neutrophils and $CD11c^+MHCII^+$ DCs from WT or $CD244^{-/-}$ mice with 1mg/ml of Ovalbumin for 24 hours and the proportion and MFI of H-2kb-$SIINFEKL^+$ population was examined by flow cytometry. To test the phagocytic ability of $CD244^{-/-}$ neutrophils and BMDCs, we co-cultured WT or $CD244^{-/-}$ neutrophils or BMDCs with carboxyfluorescein succinimidyl ester (CFSE) stained B16F10 cells. Phagocytosis against B16F10 cells was assessed by flow cytometry 24 hours later by measuring CFSE fluorescence in $Ly6C^+Ly6G^+$ neutrophils and $CD11c^+MHCII^+$ DCs. Finally, we investigated if absence of CD244 in DCs and neutrophils could affect direct activation of antigen-specific CD8T cells and hence tumor clearance. For this, we co-cultured OVA-specific CD8 T cells isolated from OT-1 mice with WT or $CD244^{-/-}$ neutrophils or DCs, in the presence of 1mg/ml of ovalbumin. IFN-γ expression of OT-1 T cells were measured 24 hours later by flow cytometry. $*P < 0.05$; unpaired Student's *t*-test **(B-C and D (right), G, H)** or two-way ANOVA **(D (left))**. Data are representative of three **(A, D, E)** or two **(C, G, H)** independent experiments or compiled from three **(B)** independent experiments.

**Fig. S4. scRNA-seq analysis and regulation of ER stress-mediated macrophage differentiation by CD244-CD48 interaction**

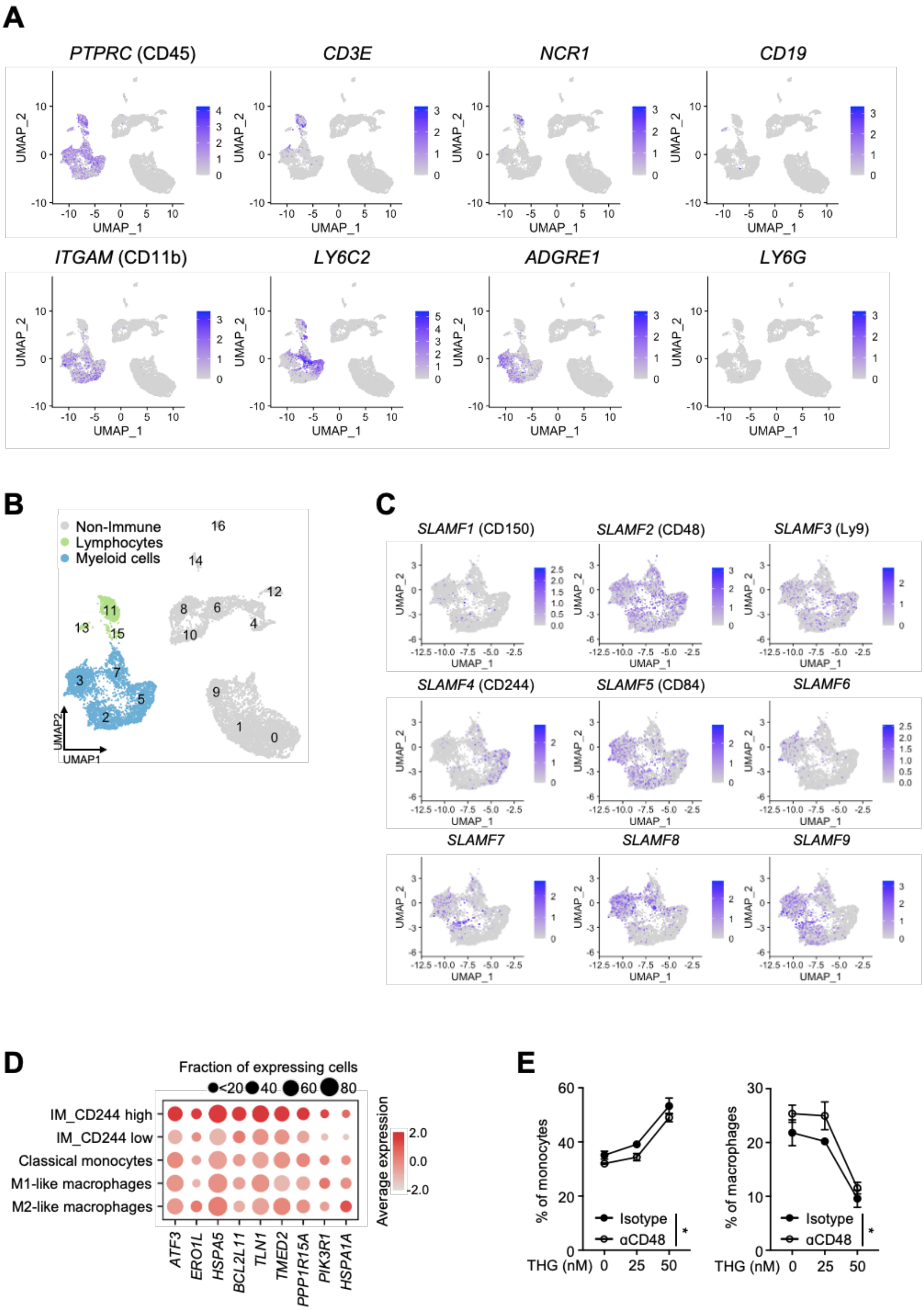

**(A-C)** The scRNA-seq data from mouse syngeneic tumors (GSE121861) was downloaded and subjected to re-analysis. **(A)** Uniform manifold approximation and projection (UMAP) plots showing immune cell markers. **(B)** A UMAP plot showing 7 immune cell clusters among a total 16 clusters, containing 3 lymphoid (11, 13, 15) and 4 myeloid (2, 3, 5, 7) clusters, along with cancer-associated fibroblasts (CAF), and tumor cells (CT26, LL2, MC-38, Sa1N, B16F10 and EMT-6). **(C)** UMAP plots showing expression of SLAMF receptors on monocyte-lineage cells. **(D)** Dotplots exhibited the expression of genes related to ER stress in CD244-high immunosuppressive monocytes (IM), CD244-low IMs, classical monocytes (CM), M1-like macrophages (M1), and M2-like macrophages (M2). **(E)** Thapsigargin (THG), an ER stress inducer, was administered to BMDMs along with M-CSF and either anti-CD48 antibody or the corresponding isotype antibody. The proportion of monocytes **(left)** and macrophages **(right)** was evaluated in WT and CD244$^{-/-}$ BMDMs. *$P < 0.05$; two-way ANOVA. Data are representative of two independent experiments.

**Fig. S5. The regulation of autophagy by CD244 in monocytes/macrophage populations**

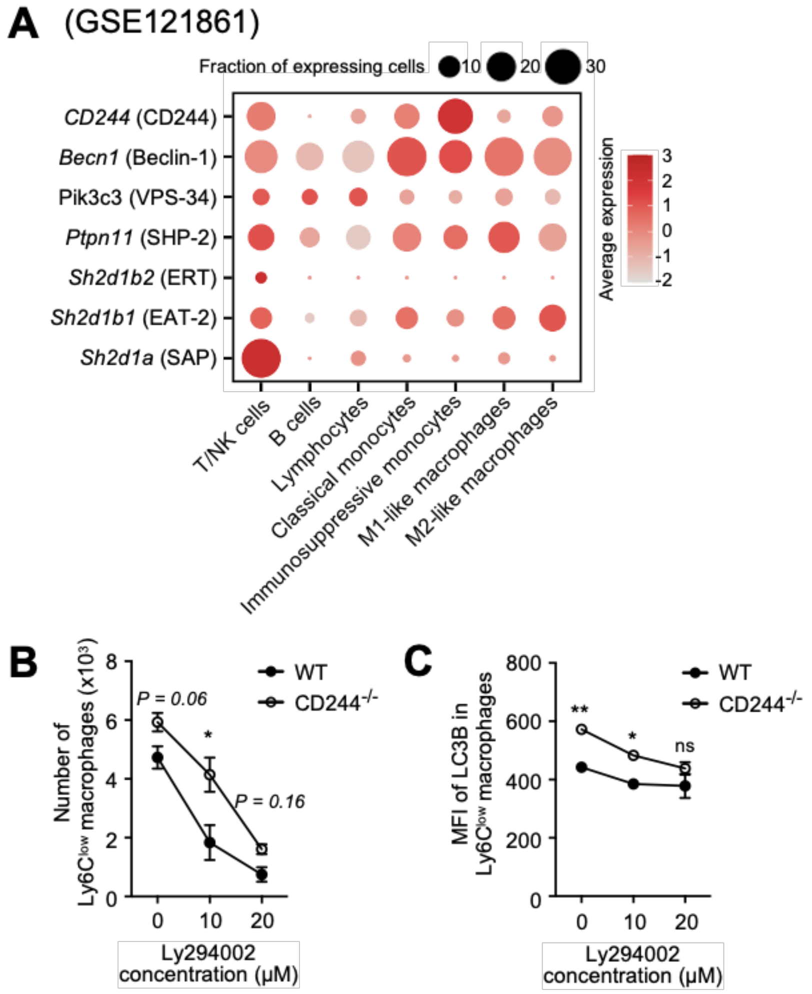


**(A)** Dotplot showing CD244 and its known adaptor molecules. **(B-C)** Number **(B)** and the LC3B expression in macrophages **(C)** were evaluated following treatment with Vps34. Vps34 serves as both an adaptor molecule of CD244 and a component of the autophagy initiation complex. *$P < 0.05$; **$P < 0.01$; ns, not significant; two-way ANOVA **(B, C)**. Data are representative of two **(B, C)** independent experiments.

**Fig. S6. Memory phenotypes of CD8/CD4 T cells in tumor of anti-PD-L1 treated CD244$^{fl/fl}$ and CD244$^{fl/fl}$LysM$^{cre}$ mice.**

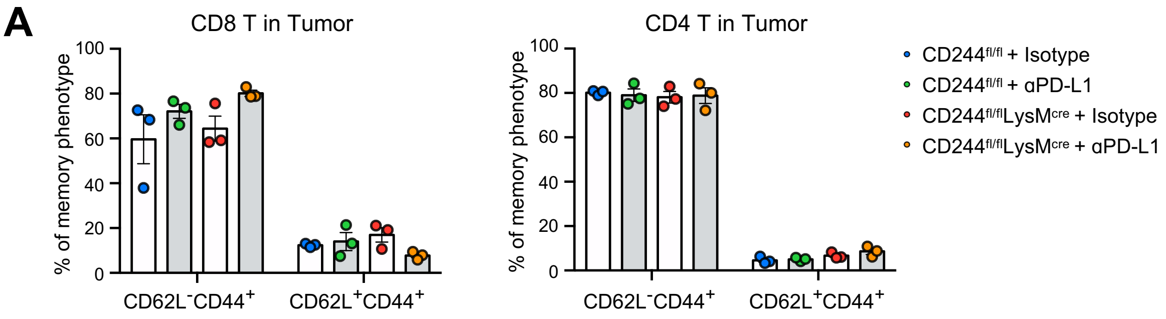


**(A)** CD244$^{fl/fl}$ and CD244$^{fl/fl}$LysM$^{cre}$ mice were treated with either anti-PD-L1 antibody or the corresponding isotype antibody 5 and 9 days after B16F10 injection. The proportion of effector memory (CD62L$^{-}$CD44$^{+}$) and central memory (CD62L$^{+}$CD44$^{+}$) in CD8 **(left)** and CD4 **(right)** T cell subsets within tumor was measured 12 days after tumor inoculation. Data are representative of two independent experiments.

**Fig. S7. The absence of CD244 led to a reduction in tumor burden in B16F10 melanoma as well as in other tumor models.**

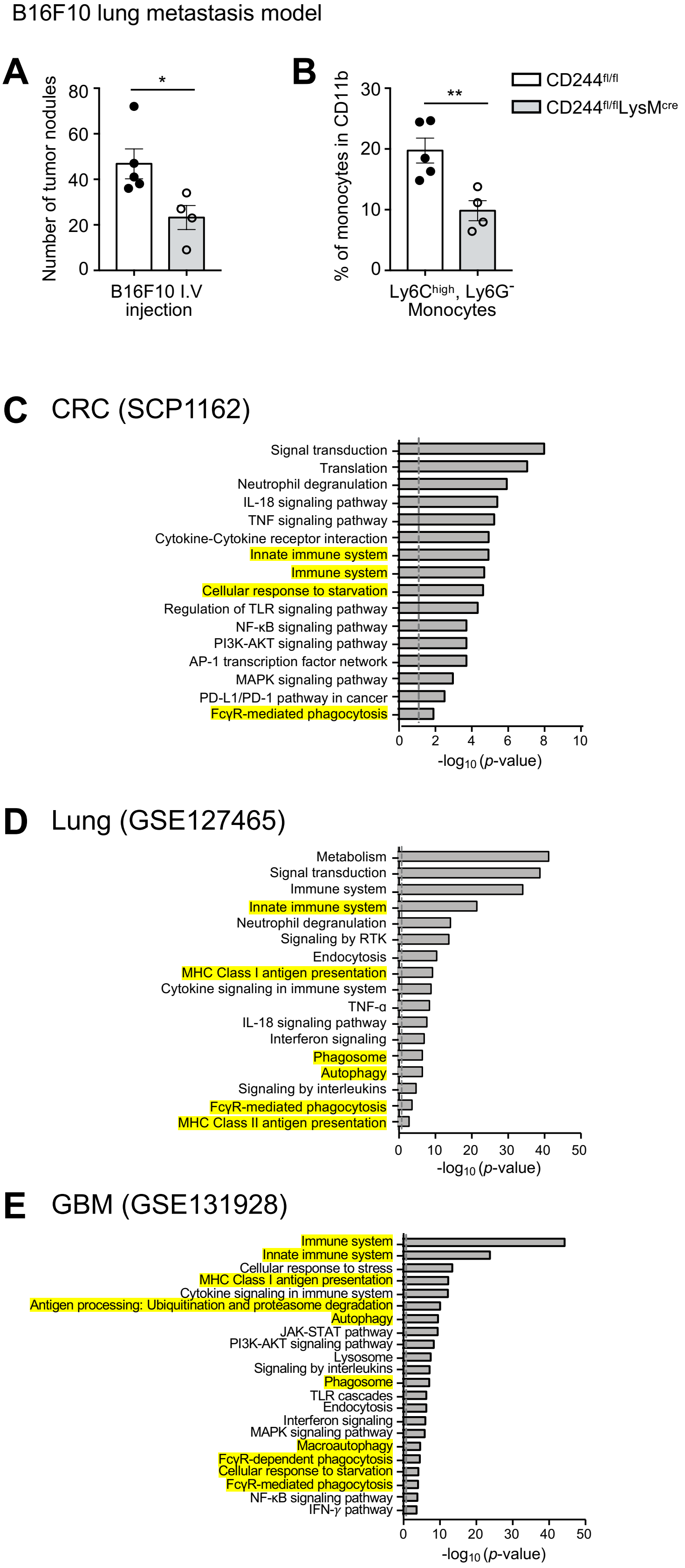

**(A-B)** The number of tumor nodules **(A)** and the proportion of monocytes **(B)** in the lungs were measured 14 days after intravenous injection of 5 x $10^5$ of B16F10 cells into CD244$^{fl/fl}$ and CD244$^{fl/fl}$LysM$^{cre}$ mice. **(C-E)** The scRNA-seq data of colorectal cancer (SCP1162) **(C)**, non-small cell lung cancer (GSE127465) **(D)** and glioblastoma (GSE131928) **(E)** were downloaded and subjected to re-analysis. Enriched signaling pathways predicted from DEGs of CD244-low monocytes/macrophages. *$P < 0.05$; **$P < 0.01$; unpaired Student's *t*-test. Data are representative of two **(A, B)** independent experiments.
